# Is sex a predictor for delayed cerebral ischaemia (DCI) and hydrocephalus after aneurysmal subarachnoid haemorrhage (aSAH)? A systematic review and meta-analysis

**DOI:** 10.1007/s00701-022-05399-0

**Published:** 2022-11-04

**Authors:** Sabah Rehman, Hoang T. Phan, Ronil V. Chandra, Seana Gall

**Affiliations:** 1 Menzies Institute for Medical Research, University of Tasmania, Hobart, TAS Australia; 2 NeuroInterventional Radiology, Monash Health, Melbourne, VIC Australia; 3 School of Clinical Sciences at Monash Health, Monash University, Melbourne, VIC Australia; 4 Monash University, Melbourne, VIC Australia

**Keywords:** Sex differences, Delayed cerebral ischaemia, Hydrocephalus, Aneurysmal subarachnoid haemorrhage

## Abstract

**Objectives:**

DCI and hydrocephalus are the most common complications that predict poor outcomes after aSAH. The relationship between sex, DCI and hydrocephalus are not well established; thus, we aimed to examine sex differences in DCI and hydrocephalus following aSAH in a systematic review and meta-analysis.

**Methods:**

A systematic search was conducted using the PubMed, Scopus and Medline databases from inception to August 2022 to identify cohort, case control, case series and clinical studies reporting sex and DCI, acute and chronic shunt-dependent hydrocephalus (SDHC). Random-effects meta-analysis was used to pool estimates for available studies.

**Results:**

There were 56 studies with crude estimates for DCI and meta-analysis showed that women had a greater risk for DCI than men (OR 1.24, 95% CI 1.11–1.39). The meta-analysis for adjusted estimates for 9 studies also showed an association between sex and DCI (OR 1.61, 95% CI 1.27–2.05). For acute hydrocephalus, only 9 studies were included, and meta-analysis of unadjusted estimates showed no association with sex (OR 0.95, 95%CI 0.78–1.16). For SDHC, a meta-analysis of crude estimates from 53 studies showed that women had a somewhat greater risk of developing chronic hydrocephalus compared to men (OR 1.14, 95% CI 0.99–1.31). In meta-analysis for adjusted estimates from 5 studies, no association of sex with SDHC was observed (OR 0.87, 95% CI 0.57–1.33).

**Conclusions:**

Female sex is associated with the development of DCI; however, an association between sex and hydrocephalus was not detected. Strategies to target females to reduce the development of DCI may decrease overall morbidity and mortality after aSAH.

**Supplementary Information:**

The online version contains supplementary material available at 10.1007/s00701-022-05399-0.

## Introduction

Delayed cerebral ischaemia (DCI) [23] and hydrocephalus [10] are the most common complications that occur following aneurysmal subarachnoid haemorrhage (aSAH). These neurological complications are also predictors of poor outcomes [27]. DCI occurs in approximately 30% of aSAH patients, mostly between 3 and 14 days after aSAH, and may lead to ischaemic stroke, and severe morbidity or mortality [29]. Hydrocephalus increases the risk of morbidity, prolongs hospital and intensive care unit stays and leads to additional neurosurgical procedures [40]. A better understanding of the sex differences in these complications, and an increase in our understanding of the potential factors that could explain any detected difference could lead to the exploration of preventative strategies to decrease overall morbidity and mortality after aSAH. Identifying sex differences has resulted in the improved management of various cardiovascular diseases eventually leading to better outcomes for both sexes [32].

Sex differences in the incidence of DCI and hydrocephalus have been explored in a few studies. In our previous multicentre observational study, we also observed that a greater proportion of women had DCI and hydrocephalus [27]. A prior systematic review published a decade ago reported limited evidence of an association between sex and DCI with the pooled odds ratio for women compared to men being OR 1.3 (95% CI 1.1–1.6) [7]. Notably, examining sex difference was not the primary objective of the previous review, and only 4 high-quality studies were included in the meta-analysis. Thus, the relationship between sex and the development of DCI remains uncertain. Identification of sex as a predictor for DCI could potentially refine current DCI prediction systems, assist in tailoring clinical and imaging surveillance for DCI and impact on the decisions to transfer patients out of intensive care into regular ward beds. Moreover, this would inform future research to examine pathophysiological pathways that could be different in men and women for the development of DCI.

For acute hydrocephalus, a systematic review with an account of sex differences could not be identified; however, for chronic shunt-dependent hydrocephalus (SDHC), results have been inconsistent. In one review which aimed to examine risk factors for SDHC after aSAH, the meta-analysis of 12 studies showed that female sex was not a risk factor for SDHC (OR 1.13, 95% CI 0.77–1.65) [40]. Another review that aimed to determine the predictors for SDHC showed that the female sex was a predictor for developing SDHC (OR 1.19, 95% CI 1.07–1.33) from the meta-analysis of 21 studies [41]. Of note, the estimates pooled in these meta-analyses were unadjusted. Like DCI, these systematic reviews on hydrocephalus were not aimed at specifically examining the sex differences. Hence, inconsistent findings in previous reviews demand an updated systematic review and meta-analysis to determine the role of sex in hydrocephalus post aSAH.

Since the relationship between sex, DCI and hydrocephalus are not well established, we aimed to examine sex differences in DCI and hydrocephalus following aSAH in a systematic review and meta-analysis. In addition, we aimed to perform multivariable modelling to increase our understanding of the potential causes of any detected sex differences.

## Methods

### Literature sources and search strategy

PubMed, Scopus and Medline via Ovid were searched from inception to August 2022. The Online Resource 1 supplementary methods provide full search strategy. Keywords and medical subject headings used for searching the databases included “sex characteristics”, “sex difference”, “gender difference” and “sex factors”. The review was registered with PROSPERO (ID: CRD42021253615). The methodology of this study is in accordance with PRISMA (Preferred Reporting Items for Systematic Reviews and Meta-Analyses) guidelines.

### Study screening for title and abstract

Two reviewers (SR and HP) screened titles and abstracts based on the following inclusion criteria: (1) cohort, case control, cross-sectional, case series or clinical trials; (2) provided estimates for sex differences in the given complications; (3) provided proportions to estimate the sex differences; (4) were published in English. Studies were excluded if they were (1) animal-based, experimental, autopsy series, or included fewer than 10 patients, or (2) in foreign languages.

### Full-text screening

For full-text screening, a study was included when meeting the following criteria: (1) being original research, either a cohort, case–control study, cross-sectional, case series or clinical trials; (2) either using alternate terms for DCI, including clinical/symptomatic vasospasm, delayed ischaemic neurological deficit (DIND), vasospasm, having a definition similar or close to the definition by the National Institute of Neurological Disorders and Stroke (NINDS) for DCI [31, 37], (3) providing effect estimates with 95% CI association of sex with the complications or raw data to calculate these. We excluded studies on angiographic vasospasm, based exclusively on cerebral infarction and those with no proper criteria to define DCI.

### Risk of bias and methodological quality assessment

Two independent reviewers (SR and HP) used the Critical Appraisal Skills Programme (CASP) [1] (Online Resource 2 Supplementary Table 1) to assess the quality of the studies. This CASP scale has a range from 0–14 for cohort (high-quality study if > 7 score), 0–12 for cross-sectional (high-quality study if > 6 score) and 0–11 for trials (high-quality study if > 6 score). Any conflict between the two reviewers was resolved by discussion. A few questions were modified for the review (Online Resource 2 See Supplementary Table 1).

### Data extraction

Reviewers (SR and HP) independently extracted predefined data items. Extracted items included author, year of publication, study period, study design, the sample size for cohort, male and female cases, follow-up period for cohort studies, mean age, effect estimates crude or adjusted for different risk factors in men and women, raw data to calculate crude estimates, measurement of DCI, covariates adjusted in the study and any potential reason provided by the authors for the identified difference between the sexes. While authors were not contacted for additional data, we included data and have provided additional sex-specific analysis from our prior publication [27, 28].

### Data analysis

This comprised of meta-analysis from published studies and separate analysis from original data from REDDISH (REducing Delays In aneurysmal Subarachnoid Haemorrhage) study due to additional data availability. We determined sex differences in the DCI and hydrocephalus in a multivariable model. The results from the REDDISH study were combined with the pooled estimates from published studies.

#### Meta-analysis of published studies

Crude and adjusted odds ratios (OR), risk ratios (RR) or hazard ratios (HR) were reported by some studies for sex differences in DCI and hydrocephalus after aSAH. The proportions of men and women provided by studies were used to calculate the unadjusted odds ratio of DCI or hydrocephalus when estimates were not provided or not calculated. We also used raw data to calculate crude estimates in women compared to men to add the study to meta-analysis when estimates were provided for men compared to women. Random-effects meta-analysis using metan command was used to pool estimates by approximating OR/RR when there were more than 2 published studies available to combine the results. Sub-group analysis was performed for comparing criteria to diagnose DCI (NINDS vs. other definitions that partly approximated gold standard criteria) and study design. Also, sensitivity analysis was performed for only studies with both unadjusted and adjusted estimates for DCI.

#### Statistical analysis for DCI and chronic hydrocephalus in REDDISH study

REDDISH was a retrospective cohort study of all patients (*n* = 575) with aSAH across two tertiary referral hospital networks in Australia from 1^st^ January 2010 to 31^st^ December 2016 [27, 28]. We used logistic regression to estimate the odds ratio (OR ± 95% confidence interval [CI]) of DCI and chronic hydrocephalus for women compared to men. We identified potential covariates to include in our model that were known to be associated with DCI and chronic hydrocephalus from ‘existing literature’ as well as analyses of study factors from the REDDISH data set (e.g. demographics, pre-stroke health or clinical factors). We used the purposeful model building to create an adjusted model selecting to include a variable in the model when (1) the covariate was associated with sex (*p*-value ≤ 0.25), (2) the covariate was associated with the outcome (*p*-value ≤ 0.25) and (3) the covariate that changed the effect of sex on the outcome by ≥ 10% [11]. The covariates added in the adjusted model for DCI included age, WFNS [World Federation of Neurosurgical Societies] (1–3 vs. 4–5), modified Fisher score (0–2 vs. 3–4), history of hypertension, smoking status (current, ex or never), location and size of the aneurysm, systolic blood pressure at presentation, extra ventricular drain placement, time to early treatment (≤ 72 h vs. > 72 h). For chronic hydrocephalus, covariates included in the model were age, WFNS (1–3 vs. 4–5), modified Fisher score (0–2 vs. 3–4), history of hypertension, location and size of the aneurysm and DCI. Then, stepwise elimination was performed to identify the factors that explained the association of sex with DCI and chronic hydrocephalus and age at ictus and modified Fisher score were forced into models. For acute hydrocephalus, there was a lack of evidence from the literature for potential covariates to create an adjusted model. Moreover, only the crude estimates were required for meta-analysis as there was only one study with adjusted estimates available across the studies for acute hydrocephalus. Analysis was performed in statistical software Stata17 (StataCorp LLC, Texas, USA) and a two-sided *p*-value < 0.05 was considered statistically significant.

#### Meta-regression

Meta-regression was performed to assess the heterogeneity for study-level factors including age, the proportion of women, the income status of the country of study (high-income vs. middle- and low-income countries) and criteria to diagnose DCI. To examine DCI as a study-level factor, the definition criteria for DCI used for diagnosis were compared between the studies that took into account similar or nearly similar criteria as NINDS to the studies that used other criteria (partly approximating with gold standard criteria) to diagnose DCI. Begg’s test was used to assess publication bias and a *p*-value < 0.05 was considered significant.

## Results

The pooled estimates comprised of results from pooling the estimates from published studies and combining the results from the REDDISH study with the meta-analysis. We included studies in the meta-analysis if they were more than two studies for the outcome. The details are discussed below.

### Meta-analysis of published studies

**Fig. 1:**
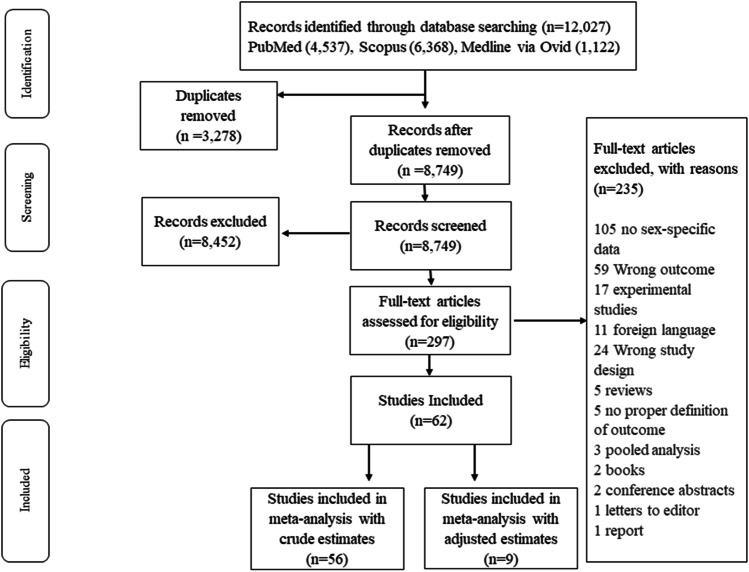
Flow diagram showing selection of eligible studies for DCI

There were 12,027 records identified for studies based on DCI and after removing duplicates there were 8749 records to screen (Fig. 1). After the screening of the title abstract and full text, 62 studies were included in the review (cohort = 25 prospective and 34 retrospective; trial = 3). There was a total of 17,061cases of aSAH in these studies. All studies were of high quality (see Supplementary Tables 2 and 3). Most of the papers were from the USA (*n* = 15), Europe (*n* = 12) and China (*n* = 11) (Online Resource 2 Supplementary Table 4).

**Fig. 2:**
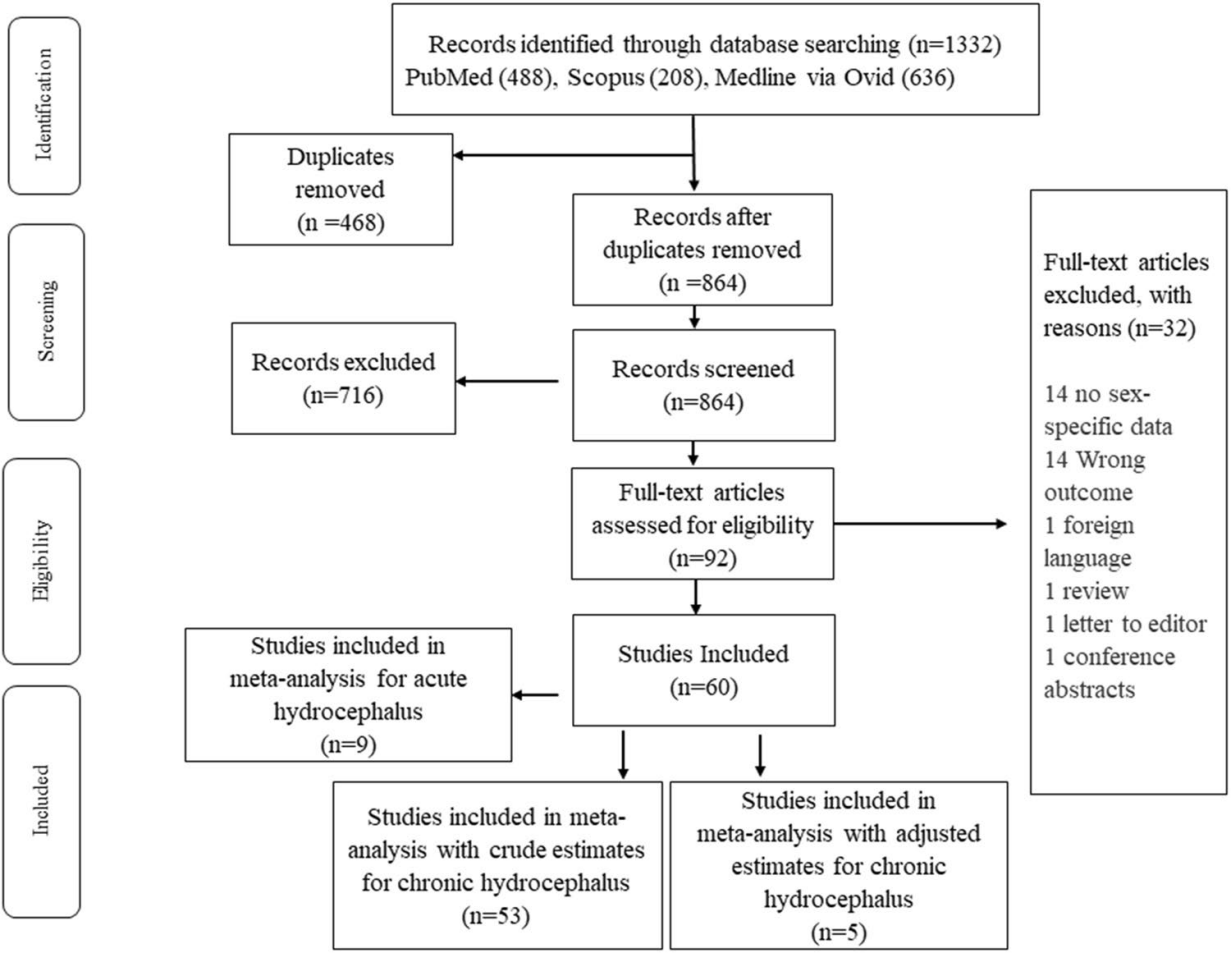
Flow diagram showing selection of eligible studies for hydrocephalus

For hydrocephalus, 1332 records were identified. After duplicates were removed (*n* = 468), we had 864 studies for the title and abstract screening (Fig. 2). Out of these, 92 studies were included in full-text screening and 60 were included in the review (cohort = 12 prospective and 45 retrospective; trials = 4). There were 31,994 cases of SAH. The majority of the included studies were of high-quality studies for hydrocephalus (see Supplementary Tables 5, 6 and 7). Most of the papers were from Europe (*n* = 17), the USA (*n* = 15) and South Korea (*n* = 10) (Online Resource 2 Supplementary Table 8).

**Fig. 3:**
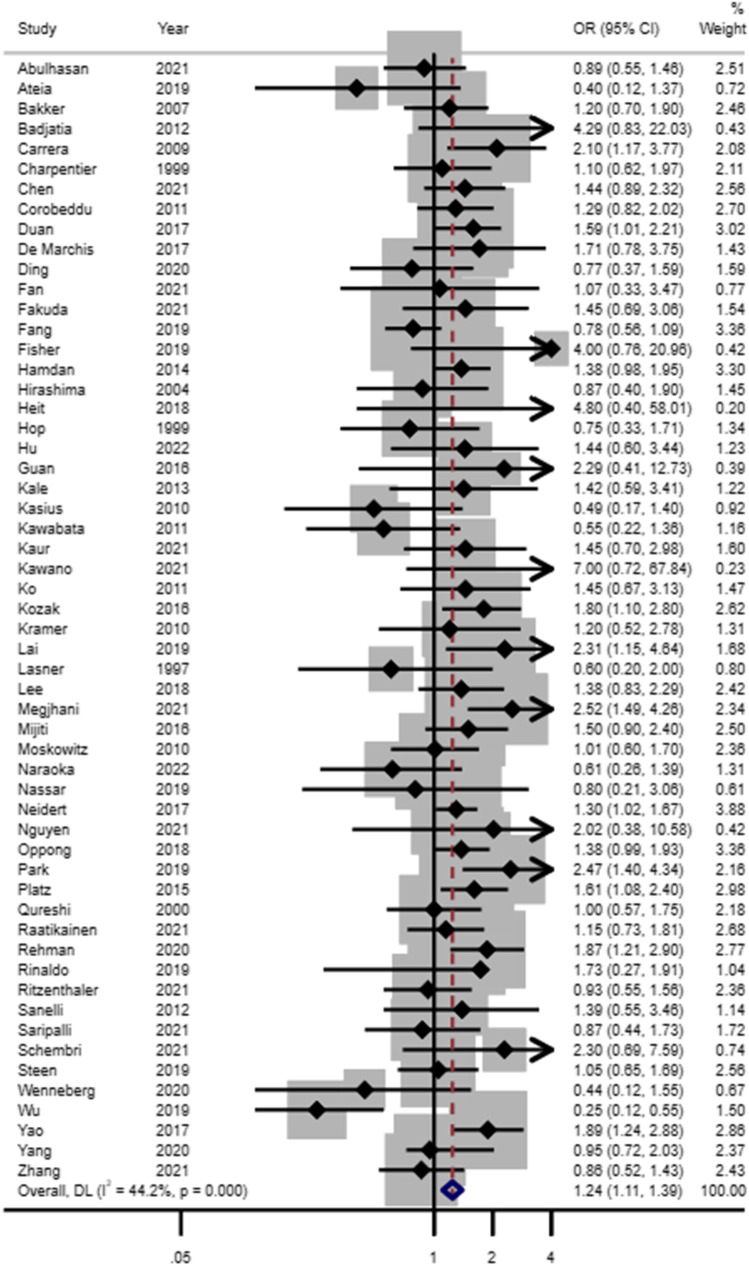
Forest plot of unadjusted estimates for DCI in women compared to men

**Fig. 4:**
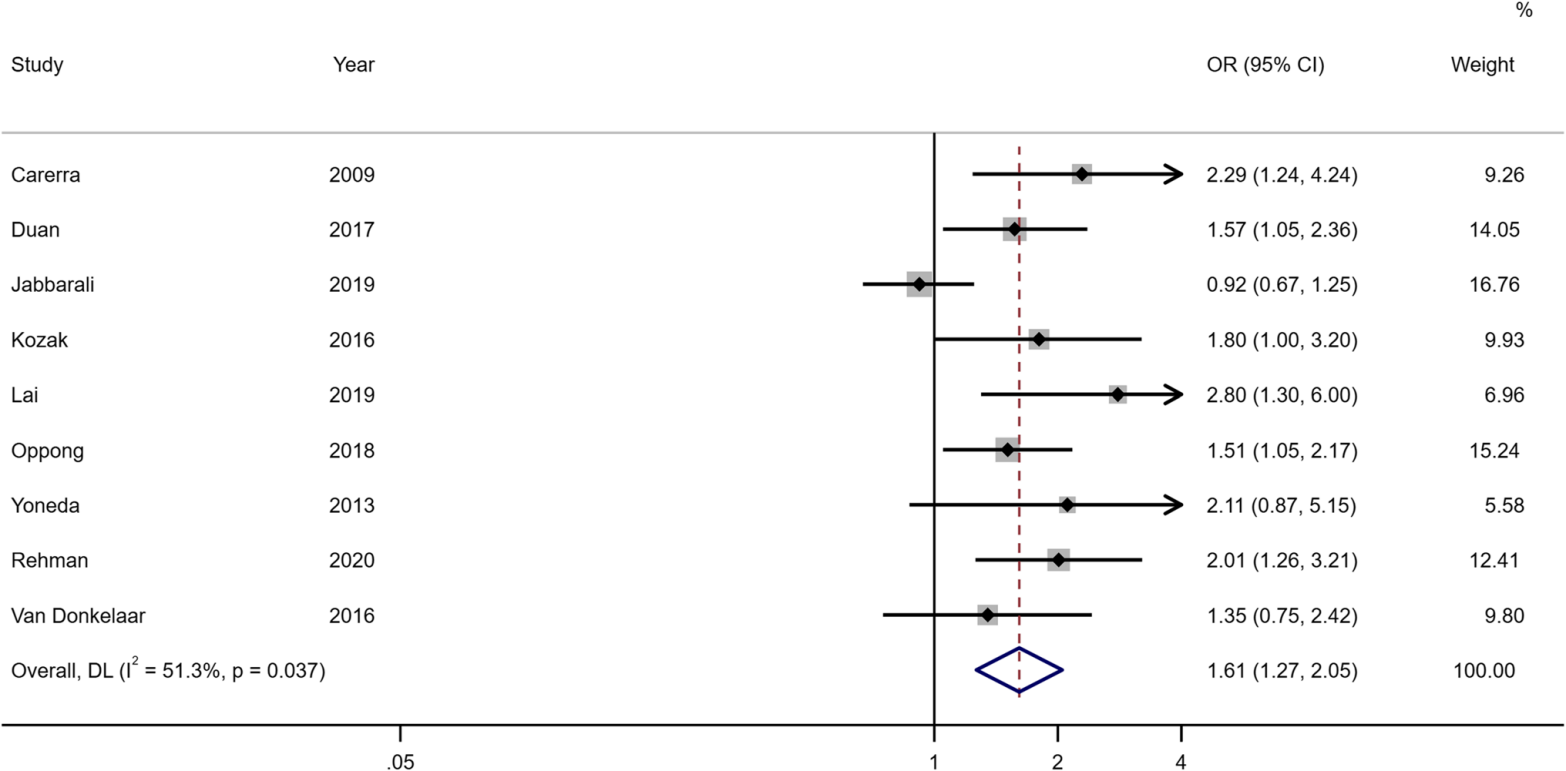
Forest plot of adjusted estimates for DCI in women compared to men

Of the 62 included studies in the DCI analysis, 56 had unadjusted women:men estimates for inclusion in our meta-analysis (Fig. 3, Online Resource 2 Supplementary Table 9). Female sex was associated with DCI in the crude meta-analysis of 56 studies (OR 1.24, 95% CI 1.11–1.39). There were approximately 15% of the included studies (*N* = 9) with adjusted estimates for DCI. Women were more likely to develop DCI compared to men even after accounting for confounding factors in the adjusted meta-analysis (OR 1.61, 95% CI 1.27–2.05) (see Fig. 4 and Online Resource 2 Supplementary Table 4). A sensitivity analysis that was limited to the studies providing both unadjusted and adjusted estimates (*N* = 6) showed consistent results (see Online Resource 2 Supplementary Fig. 1). Further analysis based on the study design also showed that DCI was associated with the female sex in the unadjusted meta-analysis of prospective studies (OR 1.35, 95% CI 1.12–1.61) and retrospective studies (OR 1.20, 95% CI 1.04–1.39) while no detectable association was noted in the meta-analysis of clinical trials (OR1.01, 95% CI 0.46–2.23). For the adjusted meta-analysis, the association with sex was noted for prospective studies (OR 1.54, 95% CI 1.05–2.26) and retrospective studies (OR 1.71, 95%CI 1.33–2.20). Two studies were not included in the meta-analysis because unadjusted estimates for men compared to women were provided that were non-significant, with OR being 1.27, (95% CI 0.48–3.35) [4] and 1.08 (95%CI 0.60–1.95) [42] respectively. In the studies with adjusted analysis, it was noticed that factors like blood pressure or diabetes mellitus were taken into account. There is a possibility that the management of these might have resulted in differences by sex in DCI. In our sub-group analysis, different definitions of DCI did not affect the association with sex. We compared the studies that followed the gold standard definition (*n* = 38 in unadjusted meta-analysis and *n* = 6 in adjusted meta-analysis) to those studies (*n* = 18 in unadjusted meta-analysis and *n* = 3 in the adjusted meta-analysis) that included part of the gold standard definition to diagnose DCI. The estimates were positively associated with the female sex using both NINDS (women:men OR_unadjusted_ 1.20, 95% CI 1.02–1.40; OR_adjusted_ 1.37, 95% CI 1.21–1.56) or other criteria for DCI (women:men OR_unadjusted_ 1.72, 95% CI 1.18–2.51; OR_adjusted_ 1.53, 95% CI 1.17–2.01) in both unadjusted and adjusted analysis.

**Fig. 5:**
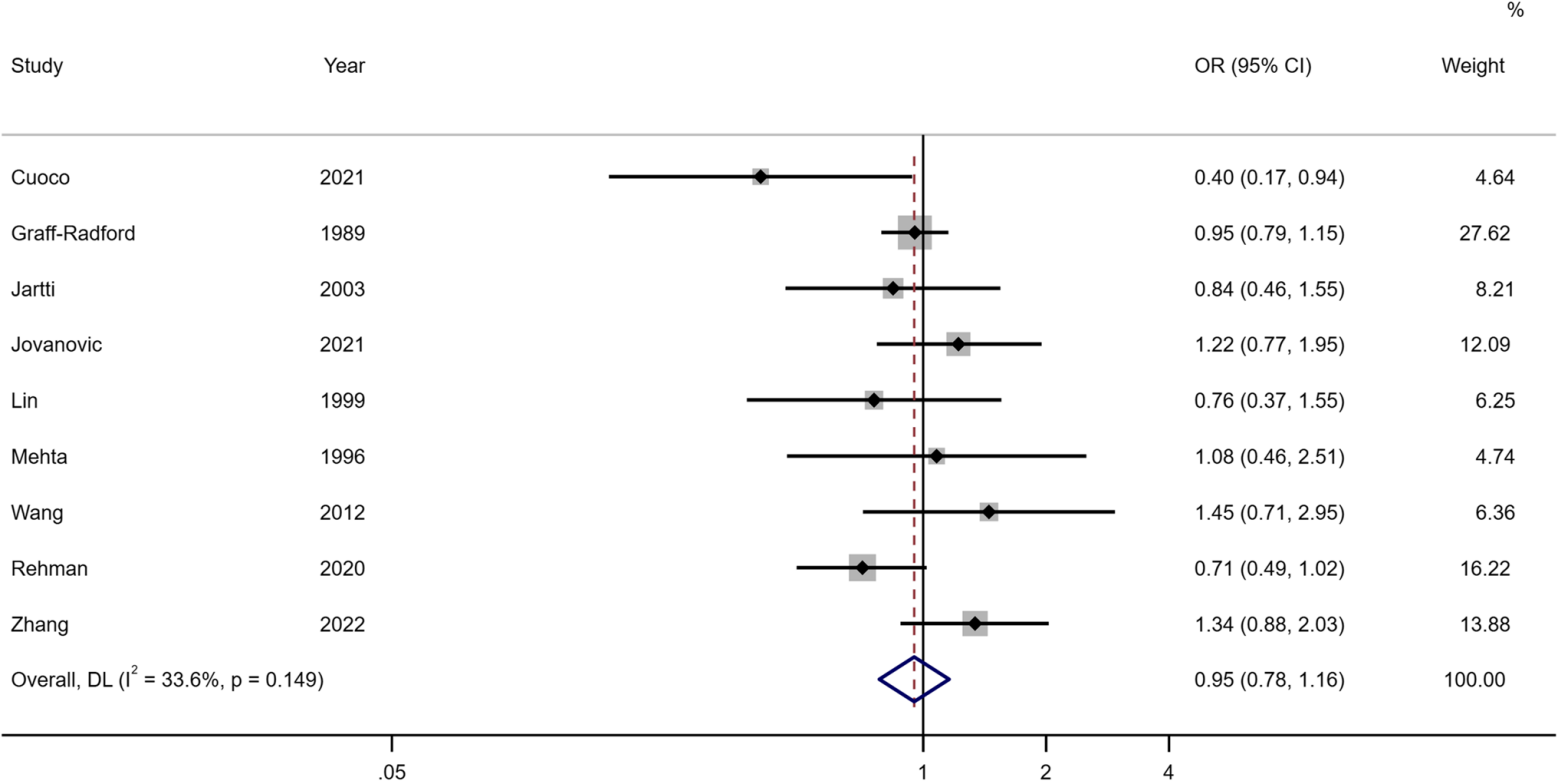
Forest plot of unadjusted estimates for acute hydrocephalus in women compared to men

**Fig. 6:**
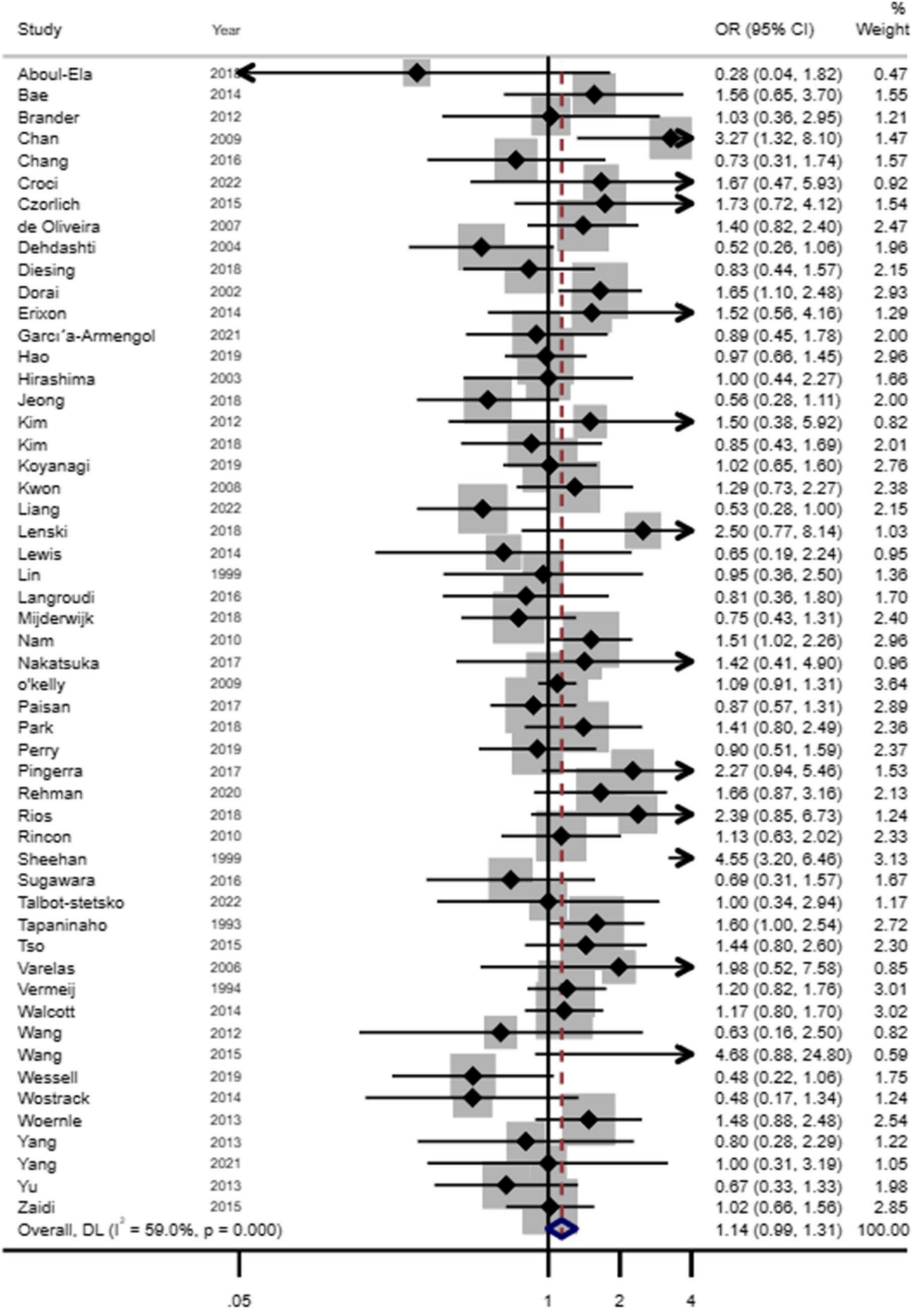
Forest plot of unadjusted estimates for chronic/SDHC in women compared to men

**Fig. 7:**
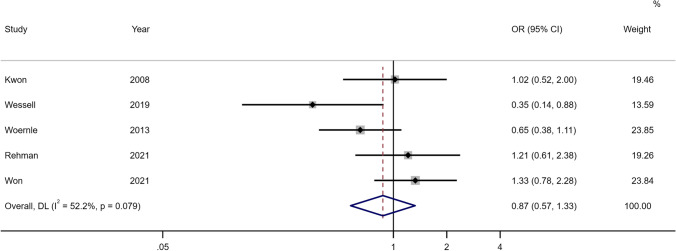
Forest plot of adjusted estimates for SDHC in women compared to men

For hydrocephalus, acute and chronic or shunt-dependent hydrocephalus were examined separately. For acute hydrocephalus, only 9 studies were included in the meta-analysis and unadjusted estimates showed no association with sex (OR 0.95, 95% CI 0.78–1.16) (see Fig. 5, Online Resource 2 Supplementary Table 10). For chronic or SDHC, the female sex was found to be somewhat of a risk in the unadjusted meta-analysis of 53 studies (OR 1.14, 95% CI 0.99–1.31) (see Fig. 6, Online Resource 2 Supplementary Table 8). There were limited studies examining the association of SDHC or chronic in the adjusted analysis. In meta-analysis for adjusted estimates from 5 studies, the risk of hydrocephalus was not associated with sex (OR 0.87, 95% CI 0.57–1.33) (see Fig. 7, Online Resource 2 Supplementary Table 8). Based on the study design, unadjusted estimates were not associated between sex and SDHC for prospective studies (OR 1.00, 95% CI 0.84–1.18), retrospective studies (OR 1.12, 95% CI 0.98–1.29) and trials (OR 1.82, 95% CI 0.78–4.24).

There was only one study with adjusted estimates for acute hydrocephalus for women compared to men (OR 0.67, 95% CI 0.18–2.43) and was not included in the meta-analysis [6]. There was one study [13] with the adjusted estimates (OR 0.5, 95% CI 0.13–10.19) for chronic hydrocephalus in men compared to women that were not significant for the male sex and not included in the meta-analysis.

### Estimates for sex differences for DCI and hydrocephalus in REDDISH study

There were 27% (*n* = 156) patients with DCI in the study and 30% (*N* = 122) women had DCI compared to 19% (*N* = 34) men. In the unadjusted analysis, sex was associated with DCI (OR 1.87, 95%CI 1.21–2.90). In a multivariable model, sex (OR 2.01, 95% CI 1.26–3.21), modified Fisher score [4, 5] (OR 2.47, 95% CI 1.24–4.93) and systolic blood pressure at presentation (OR 1.007, 95% CI 1.001–1.0013) were associated with the DCI.

Acute hydrocephalus indicated by intervention was in 55% (*n* = 315) of the patients; 61% (*n* = 108) men and 52% (*n* = 207) women. Chronic hydrocephalus was present in 10% (*n* = 59) of the aSAH patients affecting 7% (*n* = 13) men and 11% (*n* = 46) women. There was no association of sex for acute (OR 0.71, 95% CI 0.49–1.02) and chronic hydrocephalus (OR 1.66, 95% CI 0.87–3.16) in the unadjusted model. In the multivariable model, for chronic hydrocephalus, only DCI (OR 2.46, 95% CI 1.37–4.40) was an independent predictor while sex (OR 1.21, 95% CI 0.61–2.38) was not. These estimates were combined with the estimates of the published studies in the meta-analysis.

### Analysis of heterogeneity

We performed meta-regression for the factors that could potentially determine the heterogeneity among the studies. In the unadjusted meta-analysis, study characteristics including age, the income of the country, study design and criteria for DCI diagnosis were not the potential sources of heterogeneity but the greater proportion of women was a source of heterogeneity (*P* = 0.08) to some extent (see Online Resource 2 Supplementary Tables 11 and 12). In the adjusted meta-analysis for DCI, sex difference was proportional to the greater proportion of women which was identified as a potential source of heterogeneity (*p* = 0.03). For SDHC, in the unadjusted analysis, study design (trials *p* = 0.007) was observed to be the source of heterogeneity. No sources of heterogeneity were evident for studies used in the adjusted meta-analysis of SDHC and acute hydrocephalus. Also, no evidence of publication bias was detected.

## Discussion

In this systematic review, we identified sex differences for DCI in both crude and adjusted meta-analyses. No sex differences were observed for acute hydrocephalus and chronic SDHC.

### DCI

We observed that women suffer more from DCI than men even after accounting for confounders or covariates. Our findings are consistent with the previous review from almost 10 years ago that showed pooled estimates of high-quality studies to be around 1.3 (1.1–1.6) [7]. However, this review was based on examining a large number of predictors for DCI and included only 4 studies that specifically examined sex differences. By comparison, we have identified a robust association between sex and DCI in both crude analyses of 56 studies and adjusted analysis of 9 studies. The most common factors included in models with sex were age, and severity scores including WFNS, modified Fisher score or Fisher score, and Hunt and Hess score. Most of these are independent predictors of poor outcomes [25, 35], and thus our adjusted analysis adds to the current literature by demonstrating that women are approximately 1.6 times more likely to experience DCI than men.

There are many possible reasons why women more often suffer from DCI than men. An important one could be that aSAH incidence increases in women usually after menopausal age [26] when there is a decrease in oestrogen levels [24]. Oestrogen is a vasorelaxant and neuroprotective [21, 36]. It is known to increase nitric oxide (NO) bioavailability, and also regulates the prostacyclin and thromboxane A2 that maintain the vascular tone in females [22]. Oestrogen deficiency, which may contribute to aSAH in the first place [15], could lead to loss of vasodilatory effects and resultant ischaemia in women. In support of this hypothesis, there are animal studies that suggest that steroids including estradiol could be useful to relieve cerebral vasospasm [3, 18]. However, a recent study on humans with aSAH showed only a weak correlation between hormones and vascular tone [19]. More evidence is needed to assess the role of oestrogens in humans after aSAH. Another physiological phenomenon that could be the cause of DCI being more common in women than men is narrow vessel diameters with a high speed of blood flow [9]. This may cause high sheer wall stress leading to endothelial damage, lack of blood circulation and ultimately ischaemia [9]. There is still a need to understand why DCI occurs in women more often than in men and further exploration of the role of the use of oestrogen in the prevention of DCI in trials keeping in mind its implications for the risk for other cardiovascular events [34]. More studies are required to investigate the modifiable factors (e.g. pathophysiology or management) that explain the sex differences for DCI. This will refine current clinical and imaging monitoring strategies for DCI potentially leading to more tailored monitoring in women. Women at higher risk of DCI should have more frequent monitoring, and perhaps remaining in the intensive care or high dependency unit for the high-risk DCI period. Patients at lower risk could have less frequent monitoring or could be stepped down to a regular ward bed or discharged more rapidly.

The studies included in meta-analyses were examined for potential sources of heterogeneity which could be due to different study designs, sample sizes, the proportion of women, age, the income of the country and criteria to diagnose DCI. The proportion of women which remains usually high for aSAH explained variability between the studies with the adjusted estimates and to some extent for unadjusted estimates; however, other characteristics could not explain these differences. There might be unmeasured factors that could have contributed to the heterogeneity.

It was noted that there was a lack of uniformity in assessing DCI even in studies published after the introduction of gold standard criteria based on the definition of DCI by Vergouwen et al. in 2010 [37]. This lack of consistency has been highlighted in a recent study [33] that showed variations exist among neurosurgeons, intensivists and neurologists regarding the definition, diagnosis and management of DCI, and there was a ‘mediocre adherence’ to the gold standard criteria which could lead to either over or underestimation of the disease and perhaps poor care. Therefore, there is a need to take proper measures in the disparity of care of aSAH patients with DCI.

### Hydrocephalus

In our review, we found that there is somewhat a greater risk of SDHC in women than men after aSAH, but this was not associated with sex in both unadjusted or adjusted meta-analyses. We also found that there was no difference by sex for acute hydrocephalus in the crude meta-analysis.

In the previous reviews, sex was examined as a predictor for SDHC. One review showed there was no difference in the risk of SDHC in women [40] compared to men while another showed that women developed SDHC more than men [41]. In these reviews, the authors only pooled the unadjusted estimates or used raw numbers. We found few studies that provided information on sex differences in acute hydrocephalus. However, no difference was noted in the unadjusted meta-analysis. Common covariates adjusted for the studies to predict chronic or SDHC were sex, age, severity scores, acute hydrocephalus and vasospasm. As would be expected, studies showed that many patients with acute hydrocephalus often suffer from chronic hydrocephalus making it a strong predictor for SDHC [17, 20, 39]. It is observed that acute and chronic hydrocephalus share similar but also distinct pathogenesis [5], which suggests that acute hydrocephalus could be in the path of development of chronic hydrocephalus. However, acute hydrocephalus is predominantly due to obstruction of the ventricles while [14] chronic is due to obstruction of the arachnoid granulations by blood products and adhesions within the ventricular system [16]. At present, no sex differences were detected in acute hydrocephalus; however, some evidence from experimental studies suggests that acute hydrocephalus is more common in the female sex than in males and the contribution of oestrogen deficiency could potentially play the main role [12, 30]. Though we identified that the risk of developing SDHC is marginally greater in women than men further exploration is recommended to understand the relationship between sex and acute and chronic hydrocephalus as this may help in devising sex-specific measures to prevent both acute and chronic hydrocephalus and morbidity in the aftermath. As there have been very few studies specifically designed to examine sex differences in hydrocephalus with an exploration of only a limited range of covariates, therefore, further studies may help elucidate the relationship between sex and hydrocephalus.

Sex disparities have been identified in outcomes after aSAH with women having worse outcomes including mortality, functional outcomes and post-aSAH complications compared to men [2, 27]. Examining sex differences in neurological complications was not the main objective of the majority of the studies included in this review which meant most did not include sex as a covariate in multivariable models. Further exploration of the factors contributing to sex differences in complications in multivariable models will be useful to improve diagnosis and disease management [32]. Sex differences in the management of aSAH could be a potential reason for the high rate of complications in women. In the REDDISH study [28], we did not detect sex differences in adherence to care processes after aSAH. However, not many studies have been designed to compare the difference by sex in the management of aSAH; thus, lack of evidence exists as far as sex inequalities in treatment are concerned. Disregarding sex as a risk factor for DCI and hydrocephalus could potentially compromise the accuracy of management of aSAH. Adequate monitoring in women could help in early detection and timely intervention to reverse DCI as rapidly as possible before the ischemic process progresses to infarction [8], leading to worse outcomes. Therefore, it is highly recommended that future research should explore the missing link in the development of these complications in women, which may include vascular structure, hormones or genetic factors. Prospective studies and clinical trials with adequate sample size to examine sex differences in aSAH management and outcomes are needed to inform sex-specific management guidelines for aSAH including complications, which currently do not exist, unlike other cardiovascular diseases [38].

This is one of the few systematic reviews that focuses on examining sex differences in DCI and hydrocephalus after aSAH. We identified common covariates contributing to the sex differences in DCI and hydrocephalus. We carefully selected the studies regarding the definition of DCI considering the NINDS criteria and the use of alternate terms used across the studies, which was challenging. We also examined sex differences in acute hydrocephalus following aSAH noting that few studies provided data on the proportion of men and women who had acute hydrocephalus.

Some limitations should be acknowledged in this review. There were several unmeasured confounding factors, for example use of hormone replacement therapy in women, parity or other sex-specific factors that could explain sex differences but could not be addressed due to lack of evidence in the literature. For the meta-analysis of adjusted estimates for DCI and chronic hydrocephalus and crude estimates for acute hydrocephalus, there were only < 10 high-quality studies, which limits the strength of evidence and examination of the source of heterogeneity among studies.

## Conclusion

Female sex is associated with the development of DCI and but an association between sex and hydrocephalus was not detected. There is a need for more research to understand the role of sex in the occurrence of DCI post-aSAH and factors that could explain these differences. An improved understanding could lead to strategies to reduce the development of DCI and decrease overall morbidity and mortality after aSAH.

## Supplementary Information

Below is the link to the electronic supplementary material.

Supplementary file1 (DOCX 18 KB)

Supplementary file2 (DOCX 873 KB)

## References

[1] https://casp-uk.net/casp-tools-checklists/.

[2] Cai Y, Liu Z, Jia C, Zhao J, Chai S, Li Z, Xu C, Zhang T, Ma Y, Ma C (2022) Comparison of sex differences in outcomes of patients with aneurysmal subarachnoid hemorrhage: a single-center retrospective study. Frontiers in Neurology 13 10.3389/fneur.2022.853513 PMC9103686 35572942

[3] Chang C-M, Su Y-F, Chang C-Z, Chung C-L, Tsai Y-J, Loh J-K, Lin C-L (2014) Progesterone attenuates experimental subarachnoid hemorrhage-induced vasospasm by upregulation of endothelial nitric oxide synthase via Akt signaling pathway. BioMed research international 2014 10.1155/2014/207616 PMC4052693 24949428

[4] Chang JJ, Triano M, Corbin MJ, Desale S, Liu A-H, Felbaum DR, Mai JC, Armonda RA, Aulisi EF (2020). Transcranial Doppler velocity and associations with delayed cerebral ischemia in aneurysmal subarachnoid hemorrhage. J Neurol Sci.

[5] Chen S, Luo J, Reis C, Manaenko A, Zhang J (2017) Hydrocephalus after subarachnoid hemorrhage: pathophysiology, diagnosis, and treatment. BioMed research international 2017 10.1155/2017/8584753 PMC5360938 28373987

[6] Cuoco JA, Guilliams EL, Klein BJ, Benko MJ, Darden JA, Olasunkanmi AL, Witcher MR, Rogers CM, Marvin EA, Patel BM (2021). Neutrophil count on admission predicts acute symptomatic hydrocephalus after aneurysmal subarachnoid hemorrhage. World Neurosurgery.

[7] de Rooij NK, Rinkel GJ, Dankbaar JW, Frijns CJ (2013). Delayed cerebral ischemia after subarachnoid hemorrhage: a systematic review of clinical, laboratory, and radiological predictors. Stroke.

[8] Francoeur CL, Mayer SA (2016). Management of delayed cerebral ischemia after subarachnoid hemorrhage. Crit Care.

[9] Germans MR, Jaja BN, de Oliviera Manoel AL, Cohen AH, Macdonald RL (2017). Sex differences in delayed cerebral ischemia after subarachnoid hemorrhage. J Neurosurg.

[10] Germanwala AV, Huang J, Tamargo RJ (2010). Hydrocephalus after aneurysmal subarachnoid hemorrhage. Neurosurg Clinics.

[11] Greenland S (1989). Modeling and variable selection in epidemiologic analysis. Am J Public Health.

[12] Guo D, Wilkinson DA, Thompson BG, Pandey AS, Keep RF, Xi G, Hua Y (2017). MRI characterization in the acute phase of experimental subarachnoid hemorrhage. Transl Stroke Res.

[13] Hirashima Y, Hamada H, Hayashi N, Kuwayama N, Origasa H, Endo S (2003). Independent predictors of late hydrocephalus in patients with aneurysmal subarachnoid hemorrhage–analysis by multivariate logistic regression model. Cerebrovasc Dis.

[14] Jartti P, Karttunen A, Jartti A, Ukkola V, Sajanti J, Pyhtinen J (2004). Factors related to acute hydrocephalus after subarachnoid hemorrhage. Acta Radiol.

[15] Koo K, Hwang S-K (2019). Relationship between estrogen hormone and rupture of cerebral aneurysm in premenopausal women. The Nerve.

[16] Kuo L-T, Huang AP-H (2021). The pathogenesis of hydrocephalus following aneurysmal subarachnoid hemorrhage. Int J Mol Sci.

[17] Lai L, Morgan MK (2013). Predictors of in-hospital shunt-dependent hydrocephalus following rupture of cerebral aneurysms. J Clin Neurosci.

[18] Lin C-L, Shih H-C, Dumont AS, Kassell NF, Lieu A-S, Su Y-F, Hwong S-L, Hsu C (2006). The effect of 17β-estradiol in attenuating experimental subarachnoid hemorrhage–induced cerebral vasospasm. J Neurosurg.

[19] Martin J, Plank E, Ulm B, Gempt J, Wostrack M, Jungwirth B, Kagerbauer SM (2021). Concentrations of estradiol, progesterone and testosterone in sefrum and cerebrospinal fluid of patients with aneurysmal subarachnoid hemorrhage correlate weakly with transcranial Doppler flow velocities. BMC Neurosci.

[20] Nakatsuka Y, Kawakita F, Yasuda R, Umeda Y, Toma N, Sakaida H, Suzuki H (2017). Preventive effects of cilostazol against the development of shunt-dependent hydrocephalus after subarachnoid hemorrhage. J Neurosurg.

[21] Nicholson CJ, Sweeney M, Robson SC, Taggart MJ (2017). Estrogenic vascular effects are diminished by chronological aging. Sci Rep.

[22] Novella S, Dantas AP, Segarra G, Medina P, Hermenegildo C (2012). Vascular aging in women: is estrogen the fountain of youth?. Front Physiol.

[23] Oka F, Chung DY, Suzuki M, Ayata C (2020). Delayed cerebral ischemia after subarachnoid hemorrhage: experimental-clinical disconnect and the unmet need. Neurocrit Care.

[24] Oppong MD, Iannaccone A, Gembruch O, Pierscianek D, Chihi M, Dammann P, Köninger A, Müller O, Forsting M, Sure U (2018). Vasospasm-related complications after subarachnoid hemorrhage: the role of patients’ age and sex. Acta Neurochir.

[25] Park S, Megjhani M, Frey H-P, Grave E, Wiggins C, Terilli KL, Roh DJ, Velazquez A, Agarwal S, Connolly ES (2019). Predicting delayed cerebral ischemia after subarachnoid hemorrhage using physiological time series data. J Clin Monit Comput.

[26] Qureshi AI, Malik AA, Saeed O, Defillo A, Sherr GT, Suri MFK (2016). Hormone replacement therapy and the risk of subarachnoid hemorrhage in postmenopausal women. J Neurosurg.

[27] Rehman S, Chandra RV, Zhou K, Tan D, Lai L, Asadi H, Froelich J, Thani N, Nichols L, Blizzard L (2020). Sex differences in aneurysmal subarachnoid haemorrhage (aSAH): aneurysm characteristics, neurological complications, and outcome. Acta Neurochir.

[28] Rehman S, Chandra RV, Lai LT, Asadi H, Dubey A, Froelich J, Thani N, Nichols L, Blizzard L, Smith K (2021). Adherence to evidence-based processes of care reduces one-year mortality after aneurysmal subarachnoid hemorrhage (aSAH). J Neurol Sci.

[29] Rowland M, Hadjipavlou G, Kelly M, Westbrook J, Pattinson K (2012). Delayed cerebral ischaemia after subarachnoid haemorrhage: looking beyond vasospasm. Br J Anaesth.

[30] Shishido H, Zhang H, Okubo S, Hua Y, Keep RF, Xi G (2016) The effect of gender on acute hydrocephalus after experimental subarachnoid hemorrhage. In: Brain Edema XVI. Springer, pp 335–339 10.1007/978-3-319-18497-5_58 26463971

[31] Suarez JI, Sheikh MK, Macdonald RL, Amin-Hanjani S, Brown RD, de Oliveira Manoel AL, Derdeyn CP, Etminan N, Keller E, Leroux PD (2019). Common data elements for unruptured intracranial aneurysms and subarachnoid hemorrhage clinical research: a national institute for neurological disorders and stroke and national library of medicine project. Neurocrit Care.

[32] Tannenbaum C, Norris CM, McMurtry MS (2019). Sex-specific considerations in guidelines generation and application. Can J Cardiol.

[33] Tjerkstra MA, Verbaan D, Coert BA, Post R, van den Berg R, Coutinho JM, Horn J, Vandertop WP (2022). Large practice variations in diagnosis and treatment of delayed cerebral ischemia after subarachnoid hemorrhage. World Neurosurgery.

[34] Tjoe B, Fell B, LeVee A, Wei J, Shufelt C (2021). Current perspective on menopause hormone therapy and cardiovascular risk. Curr Treat Options Cardiovasc Med.

[35] van Donkelaar CE, Bakker NA, Birks J, Veeger NJ, Metzemaekers JD, Molyneux AJ, Groen RJ, van Dijk JMC (2019). Prediction of outcome after aneurysmal subarachnoid hemorrhage: development and validation of the SAFIRE grading scale. Stroke.

[36] van Winden LJ, Kok M, Acda M, Dezentje V, Linn S, Shi R-Z, van Rossum HH (2021). Simultaneous analysis of E1 and E2 by LC-MS/MS in healthy volunteers: estimation of reference intervals and comparison with a conventional E2 immunoassay. J Chromatogr B.

[37] Vergouwen MD, Vermeulen M, van Gijn J, Rinkel GJ, Wijdicks EF, Muizelaar JP, Mendelow AD, Juvela S, Yonas H, Terbrugge KG (2010). Definition of delayed cerebral ischemia after aneurysmal subarachnoid hemorrhage as an outcome event in clinical trials and observational studies: proposal of a multidisciplinary research group. Stroke.

[38] Wainer Z, Carcel C, Hickey M, Schiebinger L, Schmiede A, McKenzie B, Jenkins C, Webster J, Woodward M, Sex Group GSRCtA (2020). Sex and gender in health research: updating policy to reflect evidence. Med J Aust.

[39] Wessell AP, Kole MJ, Cannarsa G, Oliver J, Jindal G, Miller T, Gandhi D, Parikh G, Badjatia N, Aldrich EF (2018). A sustained systemic inflammatory response syndrome is associated with shunt-dependent hydrocephalus after aneurysmal subarachnoid hemorrhage. J Neurosurg.

[40] Wilson CD, Safavi-Abbasi S, Sun H, Kalani MYS, Zhao YD, Levitt MR, Hanel RA, Sauvageau E, Mapstone TB, Albuquerque FC (2017). Meta-analysis and systematic review of risk factors for shunt dependency after aneurysmal subarachnoid hemorrhage. J Neurosurg.

[41] Xie Z, Hu X, Zan X, Lin S, Li H, You C (2017). Predictors of Shunt-dependent hydrocephalus after aneurysmal subarachnoid hemorrhage? A systematic review and meta-analysis. World neurosurgery.

[42] Zhao L, Cheng C, Peng L, Zuo W, Xiong D, Zhang L, Mao Z, Wu X, Jiang X, Wang P (2022) Alcohol abuse associated with increased risk of angiographic vasospasm and delayed cerebral ischemia in patients with aneurysmal subarachnoid hemorrhage requiring mechanical ventilation. Frontiers in Cardiovascular Medicine 9 10.3389/fcvm.2022.825890 PMC9127604 35620515

