# Supplementary material for: Is sex a predictor for delayed cerebral ischaemia (DCI) and hydrocephalus after aneurysmal subarachnoid haemorrhage (aSAH)? A systematic review and meta-analysis
Source: Acta Neurochir (Wien). 2022 Nov 4;165(1):199–210. doi: 10.1007/s00701-022-05399-0 (PMC9840585; doi:10.1007/s00701-022-05399-0)
Supplement: Supplementary file 1 — Supplementary file1 (DOCX 18 KB) [file 701_2022_5399_MOESM1_ESM.docx]

**Supplementary Methods**

**Search strategy**

**Search strategy**

**PUBMED**

**DCI**

(sex characteristics[MeSH Terms]) OR sex characteristic*[Title/Abstract]) OR gender difference*[Title/Abstract]) OR sex difference*[Title/Abstract]) OR sex based[Title/Abstract]) OR sex factors[Title/Abstract]) OR sexual dimorphism[Title/Abstract]) OR female[Title/Abstract]) OR male[Title/Abstract]) OR ((men[Title/Abstract] OR man[Title/Abstract]))) OR ((women[Title/Abstract] OR woman[Title/Abstract])))))))))))))))) AND (((((((((((((subarachnoid hemorrhage[MeSH Terms]) OR ((Aneurysmal subarachnoid hemorrhage[Title/Abstract] OR Aneurysmal subarachnoid haemorrhage[Title/Abstract]))) OR aSAH[Title/Abstract]) OR Ruptured cerebral aneurysm*[Title/Abstract]) OR Ruptured brain aneurysm*[Title/Abstract]) OR Ruptured intracranial aneurysm*[Title/Abstract])))))))))))))))) AND ((((((((((((((((delayed cerebral ischemia[Title/Abstract]) (delayed cerebral ischaemia[Title/Abstract])) OR (delayed ischemic neurological deficit[Title/Abstract])) OR (delayed ischaemic neurological deficit[Title/Abstract])) OR (delayed ischemic neurological deterioration[Title/Abstract])) OR (delayed ischaemic neurological deterioration[Title/Abstract])) OR (DCI[Title/Abstract]) ) OR (DIND[Title/Abstract])) OR (delayed neurological deficit[Title/Abstract])) OR (secondary cerebral ischemia [Title/Abstract])) OR (secondary cerebral ischaemia [Title/Abstract])) OR (vasospasm[Title/Abstract])) OR (cerebral infarction[Title/Abstract])) OR (delayed cerebral infarction[Title/Abstract])) OR (symptomatic vasospasm[Title/Abstract])) OR (delayed cerebral deficit[Title/Abstract])) OR (cerebral vasospasm[Title/Abstract])

**Hydrocephalus**

(sex characteristics[MeSH Terms]) OR sex characteristic*[Title/Abstract]) OR gender difference*[Title/Abstract]) OR sex difference*[Title/Abstract]) OR sex based[Title/Abstract]) OR sex factors[Title/Abstract]) OR sexual dimorphism[Title/Abstract]) OR female[Title/Abstract]) OR male[Title/Abstract]) OR ((men[Title/Abstract] OR man[Title/Abstract]))) OR ((women[Title/Abstract] OR woman[Title/Abstract])))))))))))))))) AND (((((((((((((subarachnoid hemorrhage[MeSH Terms]) OR ((Aneurysmal subarachnoid hemorrhage[Title/Abstract] OR Aneurysmal subarachnoid haemorrhage[Title/Abstract]))) OR aSAH[Title/Abstract]) OR Ruptured cerebral aneurysm*[Title/Abstract]) OR Ruptured intracranial aneurysm*[Title/Abstract])))))))))))))))) AND (((((((((ventriculoperitoneal shunt[MeSH Terms]) OR (ventriculoperitoneal shunt*[MeSH Terms]))) OR (shunt dependent hydrocephalus[Title/Abstract])) OR (shunt dependency hydrocephalus[Title/Abstract])) OR (cerebrospinal fluid shunt[Title/Abstract])) OR (extra ventricular drain[Title/Abstract])) OR (extra ventricular drainage[Title/Abstract])) OR (acute hydrocephalus[Title/Abstract])) OR (chronic hydrocephalus[Title/Abstract])

**SCOPUS**

**DCI**

( INDEXTERMS ( "sex characteristics" )  OR  INDEXTERMS ( "sex difference" )  OR  TITLE-ABS-KEY ( "sex characteristic*" )  OR  TITLE-ABS-KEY ( "sex difference*" )  OR  TITLE-ABS-KEY ( "gender difference*" )  OR  TITLE-ABS-KEY ( "sex based" )  OR  TITLE-ABS-KEY ( "sex factors" )  OR  TITLE-ABS-KEY ( "sexual dimorphism" )  OR  TITLE-ABS-KEY ( "female*" )  OR  TITLE-ABS-KEY ( "male*" )  OR  TITLE-ABS-KEY ( "man" )  OR  TITLE-ABS-KEY ( "men" )  OR  TITLE-ABS-KEY ( "wom?n" )  AND  INDEXTERMS ( "subarachnoid hemorrhage" )  OR  TITLE-ABS-KEY ( "Aneurysmal subarachnoid hemorrhage" )  OR  TITLE-ABS-KEY ( "aSAH" )  OR  TITLE-ABS-KEY ( "Aneurysmal subarachnoid haemorrhage" )  OR  TITLE-ABS-KEY ( "Ruptured cerebral aneurysm*" )  OR  TITLE-ABS-KEY ( "Ruptured intracranial aneurysm*" )  AND  TITLE-ABS-KEY ( "delayed cerebral ischemia" )  OR  TITLE-ABS-KEY ( "delayed cerebral ischaemia" )  OR  TITLE-ABS-KEY ( "delayed ischemic neurological deficit" )  OR  TITLE-ABS-KEY ( "delayed ischaemic neurological deficit" )  OR  TITLE-ABS-KEY ( "delayed ischemic neurological deterioration" )  OR  TITLE-ABS-KEY ( "delayed ischaemic neurological deterioration" )  OR  TITLE-ABS-KEY ( "DCI" )  OR  ( "DIND" )  OR  TITLE-ABS-KEY ( "delayed neurological deficit" )  OR  TITLE-ABS-KEY ( "secondary cerebral ischemia" )  OR  TITLE-ABS-KEY ( "secondary cerebral ischaemia" )  OR  TITLE-ABS-KEY ( "vasospasm" )  OR  TITLE-ABS-KEY ( "cerebral infarction" )  OR  TITLE-ABS-KEY ( "delayed cerebral infarction" )  OR  TITLE-ABS-KEY ( "symptomatic vasospasm" )  OR  TITLE-ABS-KEY ( "delayed cerebral deficit" )  OR  TITLE-ABS-KEY ( "cerebral vasospasm" ) )

**Hydrocephalus**

( INDEXTERMS ( "sex characteristics" )  OR  INDEXTERMS ( "sex difference" )  OR  TITLE-ABS-KEY ( "sex characteristic*" )  OR  TITLE-ABS-KEY ( "sex difference*" )  OR  TITLE-ABS-KEY ( "gender difference*" )  OR  TITLE-ABS-KEY ( "sex based" )  OR  TITLE-ABS-KEY ( "sex factors" )  OR  TITLE-ABS-KEY ( "sexual dimorphism" )  OR  TITLE-ABS-KEY ( "female*" )  OR  TITLE-ABS-KEY ( "male*" )  OR  TITLE-ABS-KEY ( "man" )  OR  TITLE-ABS-KEY ( "men" )  OR  TITLE-ABS-KEY ( "wom?n" )  AND  INDEXTERMS ( "subarachnoid hemorrhage" )  OR  TITLE-ABS-KEY ( "Aneurysmal subarachnoid hemorrhage" )  OR  TITLE-ABS-KEY ( "aSAH" )  OR  TITLE-ABS-KEY ( "Aneurysmal subarachnoid haemorrhage" )  OR  TITLE-ABS-KEY ( "Ruptured cerebral aneurysm*" )  OR  TITLE-ABS-KEY ( "Ruptured intracranial aneurysm*" )  AND  INDEXTERMS ( "ventriculoperitoneal shunt" )  OR  INDEXTERMS ( "ventriculoperitoneal shunt*" )  OR  TITLE-ABS-KEY ( " shunt dependency hydrocephalus" )  OR  TITLE-ABS-KEY ( " shunt dependent hydrocephalus" )  OR  TITLE-ABS-KEY ( "cerebrospinal fluid drain" )  OR  TITLE-ABS-KEY ( "cerebrospinal fluid shunt" )  OR  TITLE-ABS-KEY ( "extra ventricular drain" )  OR  TITLE-ABS-KEY ( "acute hydrocephalus" )  OR  TITLE-ABS-KEY ( "chronic hydrocephalus" ) )

**MEDLINE**

**DCI**

1 (sex adj3 characteristic$1).ab,kf,ti.

2 (sex adj3 difference$1).ab,kf,ti.

3 (gender adj3 difference$1).ab,kf,ti.

4 Sex Characteristics/

5 sex based.ab,kf,ti.

6 sex factors.ab,kf,ti.

7 sex dimorphism.ab,kf,ti.

8 female$1.ab,kf,ti.

9 male.ab,kf,ti.

10 (men or man).ab,kf,ti.

11 wom#n.ab,kf,ti.

12 subarachnoid hemorrhage.ab,kf,ti.

13 subarachnoid hemorrhage/

14 Aneurysmal Sub-arachnoid h?emorrhage.ab,kf,ti.

15 aSAH.ab,kf,ti.

16 ruptured cerebral aneurysm$1.ab,kf,ti.

17 ruptured intracranial aneurysm$1.ab,kf,ti.

18 delayed cerebral ischemia.ab,kf,ti.

19 delayed cerebral isch?emia.ab,kf,ti.

20 delayed ischemic neurological deficit.ab,kf,ti.

21 delayed isch?emic neurological deficit.ab,kf,ti.

22 delayed ischemic neurological deterioration.ab,kf,ti.

23 delayed isch?emic neurological deterioration.ab,kf,ti.

25 DCI.ab,kf,ti.

26 DIND.ab,kf,ti.

24 delayed neurological deficit.ab,kf,ti.

25 secondary cerebral ischemia.ab,kf,ti.

26 secondary cerebral ischemia.ab,kf,ti.

27 vasospasm.ab,kf,ti.

28 cerebral infarction.ab,kf,ti.

29 delayed cerebral infarction.ab,kf,ti.

30 symptomatic vasospasm.ab,kf,ti.

31 delayed cerebral deficit.ab,kf,ti.

32 cerebral vasospasm.ab,kf,ti

33 1 or 2 or 3 or 4 or 5 or 6 or 7 or 8 or 9 or 10 or 11

34 12 or 13 or 14 or 15 or 16 or 17

35 18 or 19 or 20 or 21 or 22 or 23 or 24 or 25 or 26 or 27 or 28 or 29 or 30 or 31 or 32

36 33 and 34 and 35

**Hydrocephalus**

1 (sex adj3 characteristic$1).ab,kf,ti.

2 (sex adj3 difference$1).ab,kf,ti.

3 (gender adj3 difference$1).ab,kf,ti.

4 Sex Characteristics/

5 sex based.ab,kf,ti.

6 sex factors.ab,kf,ti.

7 sex dimorphism.ab,kf,ti.

8 female$1.ab,kf,ti.

9 male.ab,kf,ti.

10 (men or man).ab,kf,ti.

11 wom#n.ab,kf,ti.

12 subarachnoid hemorrhage.ab,kf,ti.

13 subarachnoid hemorrhage/

14 Aneurysmal Sub-arachnoid h?emorrhage.ab,kf,ti.

15 aSAH.ab,kf,ti.

16 ruptured cerebral aneurysm$1.ab,kf,ti.

17 ruptured intracranial aneurysm$1.ab,kf,ti.

18 ventriculoperitoneal shunt.ab,kf,ti.

19 ventriculoperitoneal shunt$1.ab,kf,ti.

20 shunt dependent hydrocephalus.ab,kf,ti.

21 cerebrospinal fluid shunt.ab,kf,ti.

22 extra ventricular drain.ab,kf,ti.

23 extra ventricular drainage.ab,kf,ti.

24 acute hydrocephalus.ab,kf,ti.

25 chronic hydrocephalus.ab,kf,ti.

26 1 or 2 or 3 or 4 or 5 or 6 or 7 or 8 or 9 or 10 or 11

27 12 or 13 or 14 or 15 or 16 or 17

28 18 or 19 or 20 or 21 or 22 or 23 or 24 or 25

29 26 and 27 and 28
