# Supplementary material for: Is sex a predictor for delayed cerebral ischaemia (DCI) and hydrocephalus after aneurysmal subarachnoid haemorrhage (aSAH)? A systematic review and meta-analysis
Source: Acta Neurochir (Wien). 2022 Nov 4;165(1):199–210. doi: 10.1007/s00701-022-05399-0 (PMC9840585; doi:10.1007/s00701-022-05399-0)
Supplement: Supplementary file 2 — Supplementary file2 (DOCX 873 KB) [file 701_2022_5399_MOESM2_ESM.docx]

Supplementary Table-1 Quality assessment questions derived from CASP Critical Appraisal Skills Programme (CASP)

|  | **Cohort design** | **Cross-sectional design** | **Trial** |
| --- | --- | --- | --- |
| 1 | 1.Did the study address a clearly focused issue? | 1. Did the study address a clearly focused question / issue? | 1.Did the trial address a clearly focused issue? |
| 2 | 2.Was the cohort recruited in an acceptable way? | 2. Is the research method (study design) appropriate for answering the research question? | 2.Was the assignment of patients to treatments randomised? |
| 3 | 3.Was the exposure accurately measured to minimise bias? | 3. Is the method of selection of the participants clearly described? | 3.Were all of the patients who entered the trial properly accounted for at its conclusion? |
| 4 | 4.Was the outcome accurately measured to minimise bias? | 4. Was the sample free from  (selection)bias? | 4. Were patients, health workers and study personnel ‘blind’ to treatment? |
| 5 | 5(a) Have the authors identified all important confounding factors? | 5. Was the sample of subjects representative with regard to the population to which the findings will be referred? | 5. Were the groups similar at the start of the trial? |
| 6 | 5(b) Have they taken account of the confounding factors in the design and/or analysis? | 6. Was the sample size based on pre-study considerations of statistical power? | 6. Aside from the experimental intervention, were the groups treated equally? |
| 7 | 6(a) Was the follow up of  subjects complete enough? | 7. Were the risk factor and outcome variables measured correctly using instruments/measurements that had been trialled, piloted or published previously? | 7. Is primary outcome clearly specified, with specified outcomes and results for each outcome? |
| 8 | 6(b) Was the follow up of  subjects long enough? | 8. Were the risk factor and outcome variables measured appropriate to the aims of the study? | 8. Was estimate of treatment effect precise? |
| 9 | 7.Have they reported proportions, rate and estimates? | 9. Was the statistical significance assessed? | 9. Can the results be applied to the local population, or in your context? |
| 10 | 8.Are the results precise (look confidence intervals)? | 10. Are confidence intervals given for the main results? | 10. Were all clinically important outcomes considered? |
| 11 | 9. Do you believe the results? | 11. Were the confounding factors accounted for? | 11. Are the benefits worth the harms and costs? |
| 12 | 10. Can the results be applied to the local population? | 12. Can the results be applied to general population? |  |
| 13 | 11. Do the results of this study fit with other available evidence? |  |  |
| 14 | 12. Are the implications of  this study useful for practice? |  |  |
| Total Score | 14 | 12 | 11 |

Yes scores 1, No scores 0 and Can’t tell 0.5

Supplementary Table-2 Quality assessment score for included cohort studies for DCI

| Studies | 1 | 2 | 3 | 4 | 5a | 5b | 6a | 6b | 7 | 8 | 9 | 10 | 11 | 12 | Score=14 |
| --- | --- | --- | --- | --- | --- | --- | --- | --- | --- | --- | --- | --- | --- | --- | --- |
| Abulhasan | * | * | * | * | * | * | * | * | * | * | * | * | * | * | 14 |
| Ateia | * | * | * | * | * | * | * | * | * | 0 | 1 | 0.5 | 0.5 | * | 12 |
| Bakker | * | * | * | * | * | * | * | * | * | * | * | * | * | * | 14 |
| Badjatia (2012) | * | * | * | * | * | * | * | * | * | * | * | * | * | * | 14 |
| Carrera | * | * | * | * | * | * | * | * | * | * | * | * | * | * | 14 |
| Charpentier | * | * | * | * | * | * | * | * | * | * | * | * | * | * | 14 |
| Chang | * | * | * | * | * | * | * | * | * | * | * | * | * | * | 14 |
| Chen | * | * | * | * | * | * | 0.5 | 0.5 | * | * | * | * | * | * | 13 |
| Corobeddu | * | * | * | * | * | * | * | * | * | * | * | * | * | * | 14 |
| Duan | * | * | * | * | * | * | * | * | * | 0 | 1 | * | * | * | 13 |
| De Marchis | * | * | * | * | * | * | * | * | * | * | * | * | * | * | 14 |
| Ding (2019) | * | * | * | * | * | * | * | * | * | * | 0.5 | 0.5 | 0.5 | * | 12.5 |
| Ding (2020) | * | * | * | * | * | * | * | * | * | * | * | * | * | * | 14 |
| Fan | * | * | * | * | * | * | * | * | * | * | * | * | * | * | 14 |
| Fang | * | * | * | * | * | * | * | * | * | * | * | * | * | * | 14 |
| Fischer | * | * | * | * | 0 | 0 | * | * | * | * | * | * | * | * | 12 |
| Fakuda | * | * | * | * | * | * | * | * | * | * | * | * | * | * | 14 |
| Hamdan | * | * | * | * | * | * | * | * | * | * | * | * | * | * | 14 |
| Hirashima | * | * | * | * | * | * | * | * | * | * | * | * | * | * | 14 |
| Heit | * | * | * | * | 0 | 0 | * | * | * | * | * | * | * | * | 12 |
| Hop | * | * | * | * | * | * | * | * | * | * | * | * | * | * | 14 |
| Hu | * | * | * | * | * | * | * | * | * | * | * | * | * | * | 14 |
| Guan | * | * | * | * | 0 | 0 | * | * | * | 0.5 | * | * | 0.5 | 0.5 | 10.5 |
| Jabbarali | * | * | * | * | * | * | * | * | * | * | * | * | * | * | 14 |
| Kale | * | * | * | * | * | * | * | * | * | * | * | * | * | * | 14 |
| Kasius | * | * | * | * | * | * | * | * | * | * | * | * | * | * | 14 |
| Kawabata | * | * | * | * | * | 0 | * | * | * | 0 | 0.5 | 0.5 | * | * | 11 |
| Ko | * | * | * | * | * | * | * | * | * | * | * | * | * | * | 14 |
| Kozak | * | * | * | * | * | * | * | * | * | * | * | * | * | * | 14 |
| Kramer | * | * | * | * | * | * | * | * | * | * | * | * | * | * | 14 |
| Lai | * | * | * | * | * | * | * | * | * | * | * | * | * | * | 14 |
| Lasner | * | * | * | * | * | * | * | * | * | * | * | * | * | * | 14 |
| Lee | * | * | * | * | * | * | * | * | * | 0 | * | * | * | * | 13 |
| Li | * | * | * | * | * | * | * | * | * | * | * | * | * | * | 14 |
| Megjhani | * | * | * | * | 0.5 | 0.5 | * | * | * | * | * | * | * | * | 13 |
| Mijiti | * | * | * | * | * | * | * | * | * | * | * | * | * | * | 14 |
| Moskowitz | * | * | * | * | * | * | * | * | * | * | * | * | * | * | 14 |
| Nassar | * | * | * | * | 0 | 0 | * | * | * | 0.5 | * | * | * | * | 11.5 |
| Neidert | * | * | * | * | 0 | 0 | * | * | * | * | * | * | * | * | 12 |
| Nguyen | * | * | * | * | * | * | * | * | * | * | * | * | * | * | 14 |
| Oppong | * | * | * | * | * | * | * | * | * | * | * | 0.5 | 0.5 | * | 13 |
| Park | * | * | * | * | * | * | * | * | * | * | * | * | * | * | 14 |
| Platz | * | * | * | * | * | * | * | * | * | * | * | * | * | * | 14 |
| Qureshi | * | * | * | * | * | * | * | * | * | * | * | * | * | * | 14 |
| Raatikainen | * | * | * | * | * | * | * | * | * | * | * | * | * | * | 14 |
| Rehman | * | * | * | * | * | * | * | * | * | * | * | * | * | * | 14 |
| Rinaldo | * | * | * | * | * | * | * | * | * | * | * | * | * | * | 14 |
| Ritzenthaler | * | * | * | * | * | * | * | * | * | * | * | * | * | * | 14 |
| Sanelli | * | * | * | * | 0 | 0 | * | * | * | * | * | * | * | * | 12 |
| Saripalli | * | * | * | * | * | * | * | * | * | * | * | * | * | * | 14 |
| Schembri | * | * | * | * | * | * | * | * | * | * | * | * | * | * | 14 |
| Steen | * | * | * | * | * | * | * | * | * | * | * | * | * | * | 14 |
| Van Donkelaar | * | * | * | * | 0 | * | * | * | * | * | * | * | * | * | 13 |
| Wenneberg | * | * | * | * | 0.5 | 0.5 | * | * | * | * | * | * | * | * | 13 |
| Wu | * | * | * | * | * | * | * | * | * | * | * | * | * | * | 14 |
| Yao | * | * | * | * | * | * | * | * | * | * | * | * | * | * | 14 |
| Yang | * | * | * | * | * | * | * | * | * | * | * | * | * | * | 14 |
| Yoneda | * | * | * | * | * | * | * | * | * | * | * | * | * | * | 14 |
| Zhang | * | * | * | * | * | * | * | * | * | * | * | * | * | * | 14 |
| Zhao | * | * | * | * | * | * | * | * | * | * | * | * | * | * | 14 |

Supplementary Table-3 Quality assessment score for included clinical trials for DCI

| Studies | 1 | 2 | 3 | 4 | 5 | 6 | 7 | 8 | 9 | 10 | 11 | Total=11 |
| --- | --- | --- | --- | --- | --- | --- | --- | --- | --- | --- | --- | --- |
| Qureshi | * | * | * | * | * | * | * | 0.5 | * | * | * | 10.5 |
| Kawano | * | * | * | * | 0.5 | 0.5 | * | 0.5 | * | * | 0.5 | 9 |
| Naraoka | * | * | * | * | * | * | * | 0.5 | * | * | 0.5 | 10 |

Supplementary Table-4 Included studies for DCI

| Study | Year | Country | Study years | Study design | Cases of aSAH |
| --- | --- | --- | --- | --- | --- |
| Abulhasan[2] | 2021 | Canada | 2010-2016 | Retrospective | 322 |
| Ateia[3] | 2019 | Egypt | - | Prospective | 110 |
| Bakker[6] | 2007 | Netherlands | 2002-2006 | Prospective | 321 |
| Badjatia[4] | 2012 | USA | 2008-2010 | Prospective | 50 |
| Carrera[9] | 2009 | USA | 1996-2003 | Prospective | 441 |
| Charpentier[13] | 1999 | France | 1992-1997 | Retrospective | 244 |
| Chang[11] | 2020 | USA | 2017-2018 | Retrospective | 95 |
| Chen[14] | 2021 | China | 2015-2019 | Retrospective | 333 |
| Corobeddu[15] | 2011 | USA | 2001-2011 | Retrospective | 307 |
| De Marchis[20] | 2017 | Switzerland | 2009-2011 | Retrospective | 120 |
| Ding[24] | 2020 | China | 2017 | Prospective | 126 |
| Duan[26] | 2017 | China | ? | Prospective | 504 |
| Fan[28] | 2021 | China | 2018-2019 | Retrospective | 52 |
| Fang[29] | 2019 | China | 2014-2015 | Retrospective | 702 |
| Fischer[30] | 2019 | Austria | 2012-2015 | Retrospective | 30 |
| Fukuda[31] | 2021 | Japan | 2011-2017 | Retrospective | 197 |
| Hamdan[35] | 2014 | UK | 2005-2010 | Retrospective | 617 |
| Hirashima[39] | 2004 | Japan | 1995 -2003 | Retrospective | 145 |
| Heit[37] | 2018 | Switzerland | 2016-2017 | Retrospective | 16 |
| Hop[40] | 1999 | Netherlands | 1995-1996 | Prospective | 125 |
| Hu[41] | 2022 | China | 2019-2021 | Retrospective | 109 |
| Guan[34] | 2016 | USA | 2013-2015 | Prospective | 24 |
| Jabbarali[42] | 2019 | Germany | 2005-2012 | Prospective cohorts | 1057 |
| Kale[46] | 2013 | USA | 2007-2009 | Retrospective | 108 |
| Kasius[47] | 2010 | Netherlands |  | Retrospective | 91 |
| Kaur[48] | 2021 | USA | 2006-2015 | Retrospective | 149 |
| Kawano[50] | 2021 | Japan | - | Prospective Trial | 28 |
| Kawabata[49] | 2011 | Japan | 1999-2010 | Retrospective | 102 |
| Ko[53] | 2011 | USA | 2005-2009 | Prospective | 160 |
| Kozak[55] | 2016 | Hungary | 1987-2004 | Prospective | 457 |
| Kramer[56] | 2010 | Canada | 2003-2007 | Retrospective | 152 |
| Lai[58] | 2019 | USA | 2000-2017 | Retrospective | 328 |
| Lasner[59] | 1997 | USA | 1995-1996 | Prospective | 70 |
| Lee[60] | 2018 | Canada | 2002-2011 | Retrospective | 463 |
| Megjhani[65] | 2021 | USA | 2006-2014 | Prospective | 388 |
| Mijiti[68] | 2016 | China | 2011-2015 | Retrospective | 343 |
| Moskowitz[69] | 2010 | Canada | 1997-2004 | Retrospective | 308 |
| Naraoka[73] | 2022 | Japan | 2012-2016 | Trial | 128 |
| Nassar[74] | 2019 | Egypt | 2015-2016 | Prospective | 38 |
| Neidert[75] | 2017 | Switzerland |  | Prospective | 1321 |
| Nguyen[76] | 2021 | USA | 2016-2019 | Retrospective | 54 |
| Oppong[19] | 2018 | Germany | 2013-2016 | Restrospective | 994 |
| Park[79] | 2019 | USA | 2006-2014 | Prospective | 488 |
| Platz[83] | 2015 | Germany | 2006-2011 | Retrospective | 504 |
| Qureshi[84] | 2000 | USA | 1992-1994 | Trial | 283 |
| Raatikainen[85] | 2021 | Finland | 2010-2014 | Retrospective | 340 |
| Rehman[86] | 2020 | Australia | 2010-2016 | Retrospective | 575 |
| Rinaldo[87] | 2019 | USA | 2009-2013 | Retrospective | 161 |
| Ritzenthaler[90] | 2021 | France | 2015-2020 | Retrospective | 349 |
| Sanelli[91] | 2012 | USA | 2004-2008 | Retrospective | 96 |
| Saripalli[92] | 2021 | Australia | 2015-2020 | Prospective | 175 |
| Schembri[93] | 2021 | Netherlands | 2011 onwards | Prospective | 90 |
| Steen[99] | 2019 | Netherlands | 2011-2016 | Prospective | 369 |
| Van Donkelaar[100] | 2016 | Netherlands | 2006-2011 | Retrospective | 285 |
| Wenneberg[7] | 2020 | Sweden | 2015-2016 | Prospective | 55 |
| Wu[110] | 2019 | China | 2015 | Retrospective | 122 |
| Yao[114] | 2017 | China | 2002-2015 | Retrospective | 360 |
| Yang[112] | 2020 | China | 2016-2017 | Prospective | 201 |
| Yoneda[115] | 2013 | Japan | 2008-2012 | Prospective | 204 |
| Zhang[118] | 2021 | China | 2015-2019 | Retrospective | 439 |
| Zhao[120] | 2022 | France | 2010-2015 | Retrospective | 236 |
| Total N |  |  |  |  | 17,061 |

Supplementary Table-5 Quality assessment score for included cohort studies for Hydrocephalus

| Studies | 1 | 2 | 3 | 4 | 5a | 5b | 6a | 6b | 7 | 8 | 9 | 10 | 11 | 12 | Score=14 |
| --- | --- | --- | --- | --- | --- | --- | --- | --- | --- | --- | --- | --- | --- | --- | --- |
| Aboul-Ela | * | * | * | * | 0 | 0 | * | * | * | 0.5 | * | * | * | * | 11.5 |
| Bae | * | * | * | * | * | 0 | * | * | * | * | * | * | * | * | 13 |
| Brander | * | * | * | * | 0 | 0 | * | * | * | 0.5 | * | * | * | * | 11.5 |
| Chan | * | * | * | * | 0.5 | 0 | * | * | * | 0.5 | * | * | * | * | 12 |
| Chang | * | * | * | * | * | * | * | * | * | 0.5 | * | * | * | * | 13.5 |
| Croci | * | * | * | * | * | * | * | * | * | * | * | * | * | * | 14 |
| Cuoco | * | * | * | * | * | * | 0.5 | 0.5 | * | * | * | * | * | * | 13 |
| Czorlich | * | * | * | * | * | * | * | * | * | * | * | * | * | * | 14 |
| de Oliveira | * | * | * | * | * | * | * | * | * | * | * | * | * | * | 14 |
| Dehdashti | * | * | * | * | * | * | * | * | * | * | * | * | * | * | 14 |
| Diesing | * | * | * | * | * | * | * | * | * | * | * | * | * | * | 14 |
| Dorai | * | * | * | * | 0 | 0 | * | * | * | 0.5 | * | * | * | * | 11.5 |
| Garcı´a-Armengol | * | * | * | * | * | * | * | * | * | * | * | * | * | * | 14 |
| Graff-Radford | * | * | * | * | 0 | 0 | * | * | * | 0.5 | * | * | * | * | 11.5 |
| Hao | * | * | * | * | * | 0.5 | * | * | * | 0.5 | * | * | * | * | 13 |
| Jartti* | * | * | * | * | * | 0 | * | 0.5 | * | * | * | * | * | * | 12.5 |
| Jeong | * | * | * | * | * | * | * | * | * | * | * | * | * | * | 14 |
| Jovanovic | * | * | * | * | * | * | 0.5 | 0.5 | * | * | * | * | * | * | 13 |
| Kim (2012) | * | * | * | * | * | 0 | * | * | * | 0 | * | * | * | * | 12 |
| Koyanagi | * | * | * | * | * | * | * | * | * | * | * | * | * | * | 14 |
| Kwon | * | * | * | * | * | * | * | * | * | * | * | * | * | * | 14 |
| Lenski | * | * | * | * | 0 | 0 | * | * | * | 0.5 | * | * | * | * | 11.5 |
| Lewis | * | * | * | * | * | * | * | * | * | * | * | * | * | * | 14 |
| Lin | * | * | * | * | 0 | 0 | * | * | * | 0.5 | * | * | * | * | 11.5 |
| Liang | * | * | * | * | * | * | * | 0.5 | * | * | * | * | * | * | 13.5 |
| Langroudi | * | * | * | * | * | * | * | * | * | * | * | * | * | * | 14 |
| Mehta | * | * | * | * | * | * | * | * | * | 0.5 | * | * | * | * | 13.5 |
| Mijderwijk | * | * | * | * | * | * | * | * | * | 0.5 | * | * | * | * | 13.5 |
| Nam | * | * | * | * | * | * | * | * | * | * | * | * | * | * | 14 |
| Nakatsuka | * | * | * | * | * | * | * | * | * | 0.5 | * | * | * | * | 13.5 |
| o'kelly | * | * | * | * | * | * | * | * | * | 0.5 | * | * | * | * | 13.5 |
| Paisan | * | * | * | * | * | * | * | * | * | * | * | * | * | * | 14 |
| Park | * | * | * | * | * | * | * | * | * | * | * | * | * | * | 14 |
| Perry | * | * | * | * | * | * | * | * | * | * | * | * | * | * | 14 |
| Pingerra | * | * | * | * | * | * | 0.5 | 0.5 | * | * | * | * | * | * | 13 |
| Rehman | * | * | * | * | * | * | * | * | * | * | * | * | * | * | 14 |
| Rios | * | * | * | * | 0 | 0 | * | * | * | 0.5 | * | * | * | * | 11 |
| Rincon | * | * | * | * | * | * | * | * | * | * | * | * | * | * | 14 |
| Sheehan | * | * | * | * | * | * | * | * | * | 0.5 | * | * | * | * | 13.5 |
| Sugawara | * | * | * | * | * | * | * | * | * | 0 | * | * | * | * | 13 |
| Talbot-Stetsko | * | * | * | * | 0.5 | 0.5 | 0.5 | 0.5 | * | 0.5 | * | * | * | * | 11.5 |
| Varelas | * | * | * | * | * | * | * | * | * | * | * | * | * | * | 14 |
| Vermeij | * | * | * | * | * | * | * | * | * | * | * | * | * | * | 14 |
| Walcott | * | * | * | * | 0 | 0 | * | * | * | 0.5 | * | * | * | * | 11.5 |
| Wang (2012) | * | * | * | * | * | * | * | * | * | 0.5 | * | * | * | * | 13.5 |
| Wang (2015) | * | * | * | * | * | 0 | * | * | * | 0.5 | * | * | * | * | 12.5 |
| Wessell | * | * | * | * | * | * | * | * | * | * | * | * | * | * | 14 |
| Won | * | * | * | * | * | * | * | * | * | * | * | * | * | * | 14 |
| Woernle | * | * | * | * | * | * | * | * | * | * | * | * | * | * | 14 |
| Yang (2013) | * | * | * | * | * | 0 | * | * | * | 0.5 | * | * | * | * | 12.5 |
| Yang (2021) | * | * | * | * | * | * | * | * | * | * | * | * | * | * | 14 |
| Yu | * | * | * | * | * | * | * | * | * | 0.5 | * | * | * | * | 13.5 |
| Zaidi | * | * | * | * | * | * | * | * | * | * | * | * | * | * | 14 |
| Zhang | * | * | * | * | * | * | * | * | * | * | * | * | * | * | 14 |

Supplementary Table-6 Quality assessment score for included cross-sectional studies for Hydrocephalus

| Studies | 1 | 2 | 3 | 4 | 5 | 6 | 7 | 8 | 9 | 10 | 11 | 12 | Score=12 |
| --- | --- | --- | --- | --- | --- | --- | --- | --- | --- | --- | --- | --- | --- |
| Hirashima | * | * | * | * | * | * | * | * | * | * | * | * | 12 |
| Kim (2018) | * | * | * | * | * | * | * | * | * | 0 | 0 | * | 10 |
| Sugawara | * | * | * | * | * | * | * | * | * | 0 | * | * | 11 |
| Tapaninaho | * | * | * | * | * | * | * | * | * | 0.5 | 0.5 | 1 | 11 |
| Wostrack | * | * | * | * | * | * | * | * | * | 0 | * | 1 | 11 |

Supplementary Table-7 Quality assessment score for included clinical trials for Hydrocephalus

| Studies | 1 | 2 | 3 | 4 | 5 | 6 | 7 | 8 | 9 | 10 | 11 | Score=11 |
| --- | --- | --- | --- | --- | --- | --- | --- | --- | --- | --- | --- | --- |
| Tso | * | * | * | * | * | * | * | 0.5 | * | * | 0.5 | 10 |
| Erixon | * | * | * | * | * | * | * | 0.5 | * | * | 0.5 | 10 |

Supplementary Table-8 Included studies for Hydrocephalus

| Study | Year | Country | Study years | Study design | Cases of aSAH |
| --- | --- | --- | --- | --- | --- |
| Aboul-Ela[1] | 2018 | Egypt | 2015-2016 | Prospective | 18 |
| Bae[5] | 2014 | South Korea | 2008-2011 | Retrospective | 215 |
| Brander[8] | 2012 | Germany | 2008-2009 | Retrospective | 68 |
| Chan[10] | 2009 | USA | 2004-2006 | Retrospective | 89 |
| Chang[12] | 2016 | Taiwan | 2010-2014 | Retrospective | 91 |
| Croci[16] | 2020 | Malaysia | 2009, 2010-2018 | Retrospective and prospective | 39 |
| Cuoco[17] | 2021 | USA | 2012-2020 | Retrospective | 143 |
| Czorlich[18] | 2015 | Germany | 2011-2014 | Retrospective | 206 |
| de Oliveira[21] | 2007 | Germany | 1999-2005 | Prospective | 385 |
| Dehdashti[22] | 2004 | Switzerland | 1997-2003 | Prospective | 245 |
| Diesing[23] | 2018 | Germany | 2009-2015 | Retrospective | 225 |
| Dorai[25] | 2002 | USA | 1990-1999 | Retrospective | 718 |
| Erixon[27] | 2014 | Norway | 2005-2008 | Trial | 100 |
| Garcı´a-Armengol[32] | 2021 | Spain | 2017-2020 | Prospective | 214 |
| Graff-Radford[33] | 1989 | USA | - | Prospective | 3251 |
| Hao[36] | 2019 | China | 2014-2018 | Prospective | 845 |
| Hirashima[38] | 2003 | Japan | 1995-2000 | Retrospective | 114 |
| Jartti[43] | 2003 | Finland | 1988-1999 | Retrospective | 180 |
| Jeong[44] | 2018 | South Korea | 2005-2015 | Retrospective | 275 |
| Jovanovic[45] | 2021 | Croatia | 2013-2019 | Retrospective | 357 |
| Kim[52] | 2012 | South Korea | 2007-2010 | Retrospective | 59 |
| Kim[51] | 2018 | South Korea | 2007-2016. | Retrospective | 254 |
| Koyanagi[54] | 2019 | Canada | 2009-2016. | Prospective | 621 |
| Kwon[57] | 2008 | South Korea | 1990-2006 | Retrospective | 734 |
| Lenski[61] | 2018 | Germany | 2013-2015 | Retrospective | 63 |
| Lewis[62] | 2014 | USA | 2008-2012 | Retrospective | 91 |
| Lin[64] | 1999 | China | 1992-1997 | Retrospective | 168 |
| Liang[63] | 2022 | China | 2019-2021 | Retrospective | 524 |
| Langroudi[70] | 2016 | USA | 2012-2015 | Prospective | 135 |
| Mehta[66] | 1996 | Canada | 1989-1993 | Retrospective | 105 |
| Mijderwijk[67] | 2018 | Germany | 2012-2016 | Retrospective | 227 |
| Nam[72] | 2010 | South Korea | 2004-2009 | Retrospective | 736 |
| Nakatsuka[71] | 2017 | Japan | 2007-2015 | Retrospective | 87 |
| o'kelly[77] | 2009 | Canada | 1995-2005 | Retrospective | 3120 |
| Paisan[78] | 2017 | USA | 2000-2015 | Retrospective | 888 |
| Park[80] | 2018 | South Korea | 2007-2016 | Retrospective | 418 |
| Perry[81] | 2019 | USA | 2001–2016 | Prospective | 210 |
| Pingerra[82] | 2017 | Austria | 2009-2015 | Retrospective | 217 |
| Rehman[86] | 2020 | Australia | 2010-2016 | Retrospective | 575 |
| Rios[89] | 2018 | Germany | 2012-2014 | Retrospective | 107 |
| Rincon[88] | 2010 | USA | 1996-2002 | Prospective | 580 |
| Sheehan[94] | 1999 | USA | - | Trial | 897 |
| Sugawara[95] | 2016 | Japan | 2008-2011 | Retrospective | 116 |
| Talbot-Stetsko[96] | 2022 | USA | 2014-2021 | Retrospective | 68 |
| Tapaninaho[97] | 1993 | Finland | 1980-1990 | Retrospective | 835 |
| Tso[98] | 2015 | Canada | 2005-2006 | Trial | 413 |
| Varelas[101] | 2006 | USA | 2000-2004 | Retrospective | 183 |
| Vermeij[102] | 1994 | Netherlands | 1977-1992 | Retrospective | 660 |
| Walcott[103] | 2014 | USA | 2005-2010 | Prospective | 8889 |
| Wang[105] | 2012 | Taiwan | 2003-2005 | Retrospective | 168 |
| Wang[104] | 2015 | Taiwan | 2001–2016 | Retrospective | 28 |
| Won[108] | 2021 | South Korea | 2008-2018 | Retrospective | 514 |
| Wessell[106] | 2019 | USA | 2013-2016 | Retrospective | 244 |
| Wostrack[109] | 2014 | Germany | 2010-2012 | Retrospective | 69 |
| Woernle[107] | 2013 | Switzerland | 2005-2010 | Retrospective | 389 |
| Yang[111] | 2013 | Taiwan | 2005-2006 | Retrospective | 88 |
| Yang[113] | 2021 | Taiwan | 2013-2019 | Retrospective | 63 |
| Yu[116] | 2013 | China | 2009-2012 | Retrospective | 202 |
| Zaidi[117] | 2015 | USA | 2003-2007 | Trial | 471 |
| Zhang[119] | 2022 | China | 2012-2018 | Retrospective |  |
| Total |  |  |  |  | 31,994 |

Supplemental Table-9 Characteristics of studies included for DCI (n=17,061)

| **Study** | **Year** | **Mean/median age** | **Women (%age)** | **DCI definition** | **Crude odds ratio** | **Adjusted odds ratio** | **Adjusted covariates** |
| --- | --- | --- | --- | --- | --- | --- | --- |
| Abulhasan | 2021 | 54 | 67 | DCI (based on clinical criteria) as per NINDS | ✓ |  |  |
| Ateia | 2019 |  | 61 | (1) clinical deterioration (i.e., a new focal deficit, decrease in level of  consciousness, or both) or (2) a new infarct on CT that was not visible on  the admission or immediate postoperative scan, or both-NINDS | ✓ |  |  |
| Bakker | 2007 | 56 | 72 | DCI was defined as the occurrence of new clinical features suggestive of DCI (gradually developed focal deficits, decreased level of consciousness, or both) confirmed by a new hypodense lesion on CT compatible with the clinical features-NINDS | ✓ |  |  |
| Badjatia | 2012 | 56 | 66 | DCI was defined as either the presence of symptomatic  vasospasm or the presence of an infarction on CT scan attributable to  vasospasm.1 Symptomatic vasospasm was defined as clinical deterioration (ie, a new focal deficit, decrease in level of consciousness,  or both) in the presence of confirmed vasospasm determined by CT  angiography or cerebral angiography. Decreased level of consciousness  was defined as a 2-point drop in the Glasgow Coma Score in a  24-hour period.-NINDS | ✓ |  |  |
| Carrera | 2009 | 52 | 72 | DCI was defined as clinical deterioration (i.e., a new focal deficit, decrease in level of consciousness, or both) and/or a new infarct on CT scan that was not visible on the admission or immediate postoperative scan, when the cause was thought by the research team to be vasospasm.NINDS | ✓ | ✓ | Age, SBP, Glucose, modified Fisher grade, Hess and Hunt score, Intracerebral hematoma |
| Charpentier | 1999 | 50 | 60 | Symptomatic vasospasm was diagnosed on the  basis of a combination of (1) the development of focal neurological  signs or deterioration of the level of consciousness, or both,  occurring between 3 and 14 days after SAH and (2) an increase in  mean TCD velocities of .120 cm/s in the investigated territories. | ✓ |  |  |
| Chang | 2020 | 54 | 61 | DCI was defined as focal lesions confirmed on head imaging (CTH or MRI) that correlated with either vascular distributions or watershed zones indicating hypoperfusion If patients had no focal deficits at the end of their hospitalization, head imaging is routinely not obtained and they are not presumed to have DCI. | ✓ |  |  |
| Chen | 2021 | 59 | 62 | DCI was defined as new focal neurological impairment or a decrease of two points on the Glasgow Coma Scale, which did not appear immediately following aneurysm occlusion. | ✓ |  |  |
| Corobeddu | 2011 | 55 | 63 | DCI was defined by the presence of otherwise unexplained clinical deterioration (ie, a new focal deficit, decrease of consciousness, or both) or a new infarct on brain imaging that was not visible on the admission or on early postoperative scan.-NINDS | ✓ |  |  |
| Duan | 2017 | 57 | 85 | DCI was defined as (1) clinical deterioration, including a new focal  neurological deficit (an increase of at least 2 points on the National  Institutes of Health Stroke Scale) or a decrease in the level of consciousness  (a decline of at least 2 points on the Glasgow Coma Scale),  or both, lasting for at least 1 h, and not apparent immediately after  aneurysm occlusion, and/or (2) a new infarct on CT that was not visible  on admission or on the immediate postoperative scan; other potential  causes of clinical deterioration, such as hydrocephalus, rebleeding,  or seizure, were rigorously excluded-NINDS | ✓ | ✓ | Age, WFNS, diabetes mellitus, Hunt and Hess score |
| ,De Marchis | 2017 | 56 | 69 | symptomatic vasospasms, defined as clinical deterioration (i.e., a new focal deficit, a decrease in level of consciousness, or both) along with a vasospasm seen in the digital-subtraction angiography (DSA) (reduction in arterial diameter compared to baseline DSA) or a mean velocity in the middle cerebral artery of > 200 cm/s in transcranial ultrasound | ✓ |  |  |
|  |  |  |  |  |  |  |  |
| Ding | 2020 | 53 | 56 | DCI was diagnosed based on criteria defined in a previous study “(a) clinical deterioration (i.e., a new focal deficit, decrease in level of consciousness, or both), and/or (b) a new infarct on CT that was not visible on the admission or immediate postoperative scan and cannot be attributed to other causes by means of clinical assessment, imaging of the brain, and appropriate laboratory studies-NINDS | ✓ |  |  |
| Fan | 2021 | 53 | 57 | Clinical deterioration caused by DCI was defined as the occurrence of a new focal neurological impairment such as hemiparesis, aphasia, apraxia or hemianopia, or a deterioration of consciousness by two points on the Glasgow Coma Scale that is sustained for at least 1 h. These  symptoms cannot be apparent immediately after aneurysm treatment. Some factors that could lead to similar clinical findings, such as postoperative  hematoma, iatrogenic ischemia and/or infarction, edema, hydrocephalus, metabolic derangement, seizures, and infection were excluded.-NINDS | ✓ |  |  |
| Fang | 2019 | 56 | 62 | DCI was defined as appearing clinical vasospasm or/and delayed cerebral infarction. (a) Clinical deterioration (GCS by ≥2 points, or development of new motor deficits, which excluding other etiologies) was considered as clinical vasospasm; (b) new infarct on brain CT that was not visible on the initial CT, excluding infarctions that appeared around the aneurysm within 48 hours after aneurysm surgery or endovascular treatment, was considered as delayed cerebral infarction.-NINDS | ✓ |  |  |
| Fakuda | 2021 | 64 | 64 | We defined DCI as cerebral vasospasm–induced neurological  deterioration or brain infarction confirmed by radiological imaging without other causes (such as hydrocephalus, seizure, or infection)-NINDS | ✓ |  |  |
| Fisher | 2019 | 56 | 70 | DCI was defined as acute occurring neurological deficit fulfilling the  following criteria: (i) decrease of GCS and/or increase of the National Institutes of Health Stroke Scale of at least 2 points for ≥1 h; (ii) exclusion of other causes for neurological deterioration (including epileptic seizure, intracerebral bleeding, hydrocephalus, infection, metabolic causes), and  (iii) confirmation of hypoperfusion in a perfusion CT.NINDS |  |  |  |
| Hamdan | 2014 | 55.2 | 61 | Vasospasm was defined as neurological deterioration accompanied by the narrowing of blood vessels on angiography | ✓ |  |  |
| Hirashima | 2004 | 59 | 69 | Symptomatic vasospasm was defined as delayed neurological  deterioration, including hemiparesis, aphasia, or disturbance  of consciousness, even when transient. These deficits  were not attributable to rebleeding, hydrocephalus, metabolic  disturbance, or surgical complication. | ✓ |  |  |
| Heit | 2018 | 56 | 69 | DCI was defined as new neurological deterioration that was not evident after aneurysm treatment and that could not be attributed to aneurysm rebleeding, hydrocephalus, infection, seizure, hyponatremia, or other metabolic abnormality | ✓ |  |  |
| Hop | 1999 |  | 70 | Probable ischemia was defined as a gradual decline in the level of consciousness or a gradual development of new focal deficits or both, with no evidence for a rebleed or hydrocephalus on CT, and exclusion of other medical causes, but without hypodensity on CT scan. Definite ischemia was defined as probable ischemia, but with confirmation of infarction on CT or at autopsy. In all analyses, the proportion of patients with DCI includes both definite and probable ischemia. | ✓ |  |  |
| Hu | 2022 | 57 | 67 | (1) no other causes leading to permanent or temporary focal neurological impairment between 4 and 14 days after SAH (such as aphasia, apraxia, hemianopia, or neglect); (2) ≥2 points decrease in the Glasgow Coma Scale (either on one of its components [eye opening, verbal response, or motor response] or on the total score); (3) new cerebral infarction on head CT scans not noticeable on admission or immediately after surgery; and (4) no other causes except vasospasm, between 4 and 30 days after aSAH-NINDS | ✓ |  |  |
| Guan | 2016 | 54 | 63 | DCI was defined on the basis of previously published guidelines as either of the following: 1. “The occurrence of focal neurological impairment (such as hemiparesis, aphasia, apraxia, hemianopia, or neglect), or a decrease of at least 2 points on the Glasgow Coma Scale (either on the total score or one of its individual components [eye, motor on either side, verbal]) lasting at least 1 hour, not apparent immediately after aneurysm occlusion, that cannot be attributed to other causes by means of clinical assessment, CT or MRI [magnetic resonance imaging] scanning of the brain, and appropriate laboratory studies.” 2. “The presence of cerebral infarction on CT or MR scan of the brain within 6 weeks after SAH, or on the latest CT or MR scan made before death within 6 weeks, or proven at autopsy, not present on the CT or MR scan between 24 and 48 hours after early aneurysm occlusion, and not attributable to other causes such as surgical clipping, endovascular treatment, ventricular catheter placement, or intraparenchymal hematoma.-NINDS | ✓ |  |  |
| Jabbarali | 2019 | 54.5 | 64 | DIND was considered in patients with SAH (1) with new focal neurologic  deficit or decrease of at least 2 points on the Glasgow Coma Scale, (2) persistent for at least 1 hour, (3) which was not attributable to other causes such as rebleeding, hydrocephalus, or infections.NINDS | ✓ | ✓ | Age, sex, Hunt and Hess score, Fisher score, ruptured aneurysm, intracerebral hemorrhage, IVH treatment, CSF infection. |
| Kale | 2013 | 54 | 71 | The clinical diagnosis of symptomatic vasospasm was based on the development of focal neurologic deficits or a sudden decline in  mental state without an identifiable cause other than confirmed vasospasm | ✓ |  |  |
| Kasius | 2010 | 54 | 66 | DCI was defined as a deterioration of consciousness (decrease in GCS of 6 1 point) or focal signs that lasted for at least 1 h and could not be explained by rebleeding, acute hydrocephalus, intra-cerebral hemorrhage, epileptic seizures, or hemodynamic, respiratory, or metabolic disturbances-NINDS | ✓ |  |  |
| Kaur | 2021 | 53 | 63 | DCI was defined as: “(1) clinical deterioration (i.e., a new focal neurologic deficit, decrease in level of consciousness, or both), and/or (2) a new infarct on CT that was not visible on the admission or immediate postoperative scan which was thought by the research team to be secondary to vasospasm-NINDS | ✓ |  |  |
| Kawano | 2021 | 63 | 60 | DCI was defined previously as development of new, focal neurological signs or deterioration in level of consciousness of at least 2 points on the Glasgow Coma Scale or new infarction proven on CT/MRI after  the exclusion of procedure-related infarctions that were not attributed to other causes, such as hydrocephalus, electrolyte abnormality, or sedation, by means of clinical assessment, CT or MRI, and appropriate laboratory  studies-NINDS | ✓ |  |  |
| Kawabata | 2011 | 59 | 61 | DCI was defined as clinical deterioration and/or a new infarct on CTor MRI that was not visible on admission or on the immediate postoperative scan,  when the cause was thought to be vasospasm-NINDS | ✓ |  |  |
| Ko | 2011 | 55 | 64 | DCI was defined as clinical deterioration attributable to vasospasm (clinical vasospasm) or a new infarct on brain CT related to vasospasm that was not visible on the admission or immediate postoperative scan (new infarction attributable to vasospasm) or both-NINDS | ✓ |  |  |
| Kozak | 2016 | 47 |  | Symptomatic vasospasm was a clinical entity that includes a new, delayed focal neurological deficit or mental status decline without evidence of any  other cause in SAH patients | ✓ | ✓ | Age, sex, Fisher score, Hunt and Hess score, HTN, Anterior Circulation Aneurysm |
| Kramer | 2010 | 55 | 67 | DINDs were considered to be present only if all three of the following  criteria were met. (1) There was a change in neurological status not  attributable to another apparent cause (eg, hydrocephalus, seizure, infection, or recent use of sedatives). (2) Vascular imaging (CT, MR, or  conventional angiography) performed after the onset of symptoms was  interpreted by a neuroradiologist as demonstrating any vasospasm of at  least ‘‘moderate’’ severity (.33% narrowing in relation to earlier imaging)  in a vascular territory that would potentially explain the neurological  change. (3) Symptoms were sufficiently severe and persistent for  physicians to initiate treatment (hemodynamic augmentation and/or  endovascular therapy).NINDS | ✓ |  |  |
| Lai | 2019 | 57 | 77 | DCI was measured after review of daily clinical notes using the  criteria of Vergouwen et al and subcategorized into clinical  deterioration and cerebral infarction. Clinical deterioration is  defined as an occurrence of focal neurologic impairment,  including hemiparesis, aphasia, apraxia, hemianopia, or neglect,  or a decrease of at least 2 points on the Glasgow Coma Scale  for at least 1 hour not explained by surgical complication,  aneurysm rerupture, hydrocephalus, seizure, infection, metabolic  disturbances, or causes attributed to CT or magnetic resonance  imaging (MRI) findings. Cerebral infarction was defined as the  presence of a low-density area on CT scan or a hyperintense  area on diffusion-weighted MRI sequence in a vascular territory  within 6 weeks after aSAH or the latest CT scan or MRI performed  before death within 6 weeks.NINDS | ✓ | ✓ | Sex, Age, HTN , Aneurysm location, Hunt and Hess score, modified Fisher grade |
| Lasner | 1997 | 50 | 63 | Symptomatic vasospasm was suspected by a deterioration in a patient's neurological condition between 3 and 14 days after SAH with no other explanation (metabolic disorder, infection, hydrocephalus, or complet­ed cerebral infarction) accompanied by elevated TCD velocities | ✓ |  |  |
| Lee | 2018 | 55 | 70 | DCI was defined as a focal neurological deficit attributable to a detected vascular territory of intracranial arterial narrowing (angiographic vasospasm) in the absence of alternative causes. The deficit could not be present immediately after aneurysm treatment and included hemiparesis, hemiparesthesia, aphasia, apraxia, neglect, hemianopia, and decreased level of consciousness equaling a loss of ≥ 2 points on the Glasgow Coma Scale-NINDS | ✓ |  |  |
|  |  |  |  |  |  |  |  |
|  |  |  |  |  |  |  |  |
|  |  |  |  |  |  |  |  |
| Megjhani | 2021 | 56 | 80 | DCI was defined classically, when patients met the following criteria:  delayed neurological deterioration defined as a ≥2-point  change in Glasgow Coma Scale (GCS) or new focal neurological  deficit lasting for >1 hour and not associated with surgical  treatment or a new cerebral infarct on brain imaging that is not  attributable to any other causes.NINDS | ✓ |  |  |
| Mijiti | 2016 |  | 61 | SV was defined as the development of new focal neurological signs within 21 days after aSAH onset, including worsening headaches, stiff neck and insidious onset of confusion, or decline in level of consciousness, or focal deficits not clinically or radiographically attributable to other causes (hydrocephalus, seizures, metabolic derangement, infection, etc.) | ✓ |  |  |
| Moskowitz | 2010 | 55 | 67 | Vasospasm was defined with a combination of clinical and radiographic criteria. Clinical symptoms was defined as onset of a new focal or global neurological deficit not explained by hydrocephalus, hemorrhage,  surgical complications, fever, infections, or metabolic abnormalities | ✓ |  |  |
| Naraoka | 2022 | 60 | 62 | DCI was defined as clinical deterioration of more than two points on the Glasgow Coma Scale (GCS), the development of new, focal neurological  signs, or both, when the cause was felt to be ischemia attributable to vasospasm after other possible causes of worsening had been excluded-NINDS | ✓ |  |  |
| Nassar | 2019 | 50 | 60 | NIHSS for early detection of new focal neurological  signs of DCI | ✓ |  |  |
| Neidert | 2017 | 55 | 64 | Clinical deterioration attributable to DCI  was defined according to Vergouwen et al as a delayed decrease of  consciousness by at least 2 points on the Glasgow Coma Scale (GCS)  and/or a new focal neurological deficit, after ruling out other causes-NINDS | ✓ |  |  |
| Nguyen | 2021 | 62 | 76 | Documentation of DCI based on a neurocritical care faculty investigators’ review of the entire medical record for the admission, for the presence of otherwise unexplained clinical deterioration during the appropriate period  of risk (days 3–21 following ictus) or the appearance of delayed  infarction on imaging-NINDS | ✓ |  |  |
| Oppong | 2018 | 55 | 67 | SV was made upon the following criteria: (a) occurrence of neurological deterioration (new focal neurological deficit and/or decrease in Glasgow  Coma Scale > 1 point), (b) exclusion of other causes of neurological  worsening (such as re-bleeding or hydrocephalus) by a follow-up CT scan, (c) TCD observations suspicious for vasospasm (> 120 cm/c), (d) confirmation of vasospasm on digital subtraction angiography (DSA). Infarcts documented > 48 h after early aneurysm occlusion were defined  as delayed cerebral ischemia (DCI) as according to the definition of Vergouwen et al. | ✓ | ✓ | Age, sex, Fisher score, hydrocephalus |
|  |  |  |  |  |  |  |  |
| Park | 2019 | 54 | 68 | DCI, defined as development of new focal neurologic signs or deterioration of consciousness for > 1 hour or appearance of new infarctions on imaging due to VSP | ✓ |  |  |
| Platz | 2015 | 53.5 | 67 | DCI was defined as any new ischemic lesion on the CT or MRI before discharge compared with the imaging after aneurysm occlusion | ✓ |  |  |
| Qureshi | 2000 | 52 | 73 | a) the classic symptoms, including onset between days 5 and 12 after SAH; worsening of headache, stiff neck, or low-grade fever; insidious onset of confusion or disorientation, or decline in level of consciousness; and focal deficit, which may fluctuate in severity; b) a head CT scan that excluded other causes of neurologic worsening, such as rebleeding or hydrocephalus; and c) no other identifiable cause of neurologic worsening, such as electrolyte disturbance, hypoxia, or seizures. | ✓ |  |  |
| Raatikainen | 2021 | 56 | 60 | DCI (based on clinical criteria) as per NINDS | ✓ |  |  |
| Rehman | 2020 | 56 | 69 | DCI (based on clinical criteria) as per NINDS | ✓ | ✓ | Age, SBP at presentation, WFNS, modified Fisher grade, location of aneurysm, size of aneurysm, HTN history, smoking status, time to early treatment |
| Rinaldo | 2019 | 54 | 65 | DCI was defined as delayed neurological deterioration after aSAH not due to other causes evident on clinical, laboratory, or radiographic evaluation, as previously described-NINDS | ✓ |  |  |
| Ritzenthaler | 2021 | 54 | 65 | DCI, which was defined as any focal neurological impairment or any decrease of at least 2 points on the GCS, lasting for at least 1 h, that was not apparent immediately after aneurysm occlusion, and could not be attributed  to other causes-NINDS | ✓ |  |  |
| Sanelli | 2012 | 50 | 77 | DCI, defined as clinical deterioration with the occurrence of focal neurologic impairment (such as hemiparesis, hemiplegia, aphasia, and so forth) or a decrease of at least 2 points on the Glasgow Coma Scale that was not apparent immediately after aneurysm occlusion and was not attributed to other causes by clinical assessment, CT or MR imaging,  and laboratory studies.-NINDS | ✓ |  |  |
| Saripalli | 2021 | 55 | 71 | Clinical deterioration owing to DCI was based on a decrease in  the Glasgow Coma Scale score of 2 points or an increase in the  National Institutes of Health Stroke Scale score of 2 points lasting  for at least 1 hour-NINDS | ✓ |  |  |
| Schembri | 2021 | 52 | 70 | A new focal neurological deficit (motor or speech)  or a decrease of two points or more on the Glasgow Coma  Scale, for at least 1 h, that could not be attributed to other  causes such as hydrocephalus, electrolyte or metabolic  disturbances, re-bleeding, post-treatment complications,  meningitis, or other kinds of infections-NINDS | ✓ |  |  |
| Steen | 2019 | 57 | 68 | DCI, defined as the occurrence of new focal neurologic impairment or a decrease of >2 points on the Glasgow Coma Scale (with or without new  hypodensity on CT) that could not be attributed to other causes, in  accordance with the definition proposed by a multidisciplinary  research group-NINDS | ✓ |  |  |
| Van Donkelaar | 2016 | 55 | 66 | DCI defined as a new hypodensity on CT not otherwise  explained than by cerebral infarction due to DCI within 30  days after admission | ✓ | ✓ | Age, sex, WFNS, Hijdra score maximum lactate and glucose |
| Wenneberg | 2020 | 58 | 73 | Clinical deterioration due to DCI was defined as  focal neurological impairment or a two-point decrease in GCS score,  which lasts for at least 1 hour and is not attributed to other causes.  Cerebral infarction from DCI was defined as the presence of cerebral  infarction on CT or MRI within 6 weeks after aSAH that is not  attributed to aneurysmal surgery-NINDS | ✓ |  |  |
| Wu | 2019 | 55 | 61 | DCI was defined as the occurrence of focal impairment or a decrease in at least 2 points on the Glasgow Coma Scale, which cannot be  attributed to other causes by means of clinical assessment,  imaging, and appropriate laboratory studies-NINDS | ✓ |  |  |
| Yao | 2017 | 55 | 47 | Grades I and II were named non-symptomatic ischemia, and grades  III and IV were named symptomatic ischemia using combination of infarction and neurological dysfunction. | ✓ |  |  |
| Yang | 2020 | 55 | 55 | DCI was defined as (1) clinical deterioration (a new focal neurological deficit (at least increasing 2 points on the NIHSS score) or a decrease in the level of consciousness (at least reducing 2 points on the Glasgow Coma Scale), or both, lasting for at least 1 h, and/or (2) a new infarct on CT; other  potential causes of clinical deterioration, such as hydrocephalus-NINDS | ✓ |  |  |
| Yoneda | 2013 | 64 | 70 | DCI was also defined as symptomatic cerebral  vasospasm and cerebral infarction caused by cerebral vasospasm.NINDS |  | ✓ | Age, sex, pulmonary edema, surgical clipping, poor grade SAH, cardiac index extravascular lung water index, global end-diastolic volume index, pulmonary vascular permeability index, SVRI, systemic vascular resistance index. |
| Zhang | 2021 | 62 | 69 | DCI was described as a focal neurologic deficit in the absence of other  possible related causes and/or a decrease at least 2 scores on the  Glasgow Coma Scale-NINDS | ✓ |  |  |
| Zao | 2022 | 55 | 63 | Delayed derebral ischemia (DCI) was defined as a delayed clinical deterioration due to ischemia (focal neurological impairment or a decrease of at least 2 points on the GCS that lasts for at least 1 hour that cannot be attributed to another cause), or as a delayed cerebral infraction (infarction on CT scan or MRI scans performed within 6 weeks after SAH, absent from the scan performed between 24 and 48 hours after aneurysm occlusion, and not attributable to another cause: aneurysm-securing procedure or extraventricular derivation placement-NINDS | ✓ |  |  |

SBP: systolic blood pressure, WFNS: world federation for neurological societies, HTN: hypertension, IVH: intraventricular hemorrhage NINDS: National Institute of Neurological Disorders and Stroke

Supplementary Table-10 Characteristics of studies with Hydrocephalus (N=31,994)

| **Study** | **Year** | **Mean/median age** | **Women (%age)** | **Crude odds ratio** | **Adjusted odds ratio** | **Adjusted covariates** |
| --- | --- | --- | --- | --- | --- | --- |
| Aboul-Ela | 2018 | 54 | 50 | ✓ |  |  |
| Bae | 2014 | 56 | 65 | ✓ |  |  |
| Brander | 2012 | 52 | 68 | ✓ |  |  |
| Croci | 2020 | 60 | 54 | ✓ |  |  |
| Cuoco | 2021 | 55 | 81 | ✓ | ✓ | Ventilation on admission, Hunt and Hess grade 4-5, modified Fisher score 3-4, IVH, Neutrophil count, Monocyte count, neutrophil-to-lymphocyte ratio, monocyte-neutrophil-to-lymphocyte ratio, HTN, current smoker, prior ischemic stroke |
| Chan | 2009 |  | 64 | ✓ |  |  |
| Chang | 2016 | 57 | 59 | ✓ |  |  |
| Czorlich | 2015 | 55 | 70 | ✓ |  |  |
| de Oliveira | 2007 | 53 | 67 | ✓ |  |  |
| Dehdashti | 2004 | 49 | 62 | ✓ |  |  |
| Diesing | 2018 | 54 | 68 | ✓ |  |  |
| Dorai | 2002 | 53 | 67 | ✓ |  |  |
| Erixon | 2014 | 57 | 73 | ✓ |  |  |
| Garcı´a-Armengol | 2021 | 57 | 60 | ✓ |  |  |
| Graff-Radford* | 1989 |  | 61 | ✓ |  |  |
| Hao | 2019 | 52 | 62 | ✓ |  |  |
| Hirashima | 2003 | 58 | 67 | ✓ | ✓ | Sex, Severity scores, duration of drainage, vasospasm, vasopressor, operation time |
| Jartti* |  |  | 44 | ✓ |  |  |
| Jeong | 2018 | 71 | 80 | ✓ |  |  |
| Jovanovic | 2021 | 56 | 67 | ✓ |  |  |
| Kim | 2012 | 52 | 62 | ✓ |  |  |
| Kim | 2018 | 55 | 71 | ✓ |  |  |
| Koyanagi | 2019 | 65 | 75 | ✓ |  |  |
| Kwon | 2008 | 53 | 68 | ✓ | ✓ | Age, Hunt and Hess score, IVH, ICH, fisher, acute HC, posterior circulation, vasospasm, meningitis |
| Liang | 2022 |  | 64 | ✓ |  |  |
| Lenski | 2018 | 55 | 67 | ✓ |  |  |
| Lewis | 2014 | 59 | 63 | ✓ |  |  |
| Lin* | 1999 | 47 | 60 | ✓ |  |  |
| Langroudi | 2016 | 51 | 78 | ✓ |  |  |
| Mehta* | 1996 |  |  |  |  |  |
| Mijderwijk | 2018 | 60 | 62 | ✓ |  |  |
| Nam | 2010 | 62 | 64 | ✓ |  |  |
| Nakatsuka | 2017 | 64 | 72 | ✓ |  |  |
| o'kelly | 2009 | 55 | 65 | ✓ |  |  |
| Paisan | 2017 | 58 | 69 | ✓ |  |  |
| Park | 2018 | 55 | 32 | ✓ |  |  |
| Perry | 2019 | 56 | 63 | ✓ |  |  |
| Pingerra | 2017 | 57 | 67 | ✓ |  |  |
| Rehman* | 2020 | 56 | 69 | ✓ | ✓ | Age, DCI |
| Rios | 2018 | 57 | 55 | ✓ |  |  |
| Rincon | 2010 | 54 | 67 | ✓ |  |  |
| Sheehan | 1999 | 51 | 68 | ✓ |  |  |
| Sugawara | 2016 | 59 | 53 | ✓ |  |  |
| Talbot-Stetsko | 2022 | 56 | 73 | ✓ |  |  |
| Tapaninaho | 1993 | 51 | 47 | ✓ |  |  |
| Tso | 2015 | 52 | 71 | ✓ |  |  |
| Varelas | 2006 | 53 | 61 | ✓ |  |  |
| Vermeij | 1994 | 54 | 62 | ✓ |  |  |
| Walcott | 2014 | 58 | 59 | ✓ |  |  |
| Wang* | 2012 | 55 | 69 | ✓ |  |  |
| Wang | 2015 | 55 | 54 | ✓ |  |  |
| Wessell | 2019 | 55 | 55 | ✓ | ✓ | HTN, WFNS, modified Fisher grade, vasospasm, acute HC, SIRS |
| Won | 2021 | 55 | 51 |  | ✓ | age, Hunt & Hess, Modified Fisher score, aneurysm location, IVH, ICH, vasospasm, HTN, Diabetes, ventriculomegaly |
| Wostrack | 2014 | 57 | 65 | ✓ |  |  |
| Woernle | 2013 | 54 | 66 | ✓ | ✓ | Sex, GCS score, aneurysm coiling and clipping, Hunt and Hess score, Fisher score |
| Yang | 2013 | 56 | 71 | ✓ |  |  |
| Yang | 2021 | 63 | 71 | ✓ |  |  |
| Yu | 2013 | 54 | 61 | ✓ |  |  |
| Zaidi | 2015 | 53 | 70 | ✓ |  |  |
| Zhang | 2022 | 54 | 63 | ✓ |  |  |

*Studies with raw data on Acute HC; abbreviations HTN: hypertension, IVH: intraventricular hemorrhage, ICH, intracerebral hemorrhage, SIRS: Systemic inflammatory response syndrome, WFNS: world federation for neurological societies

Supplementary Table-11 Analysis of Heterogeneity for DCI studies

|  | **No. of studies** | **OR (95% CI)** | **P-value** |
| --- | --- | --- | --- |
| **DCI**  **(Unadjusted analysis)** |  |  |  |
| **Age** | 53 | 0.98 (0.94-1.03) | 0.53 |
| **Women (%)** | 55 | 1.01 (0.99-1.02) | 0.08 |
| **High income vs Middle/Low income countries** |  |  |  |
| High | 54 | Ref |  |
| Middle/Low | 2 | 0.43 (0.15-1.25) | 0.12 |
| **Study design** |  |  |  |
| Prospective | 21 | Ref |  |
| Retrospective | 34 | 0.87 (0.69-1.15) | 0.39 |
| Trial | 6 | 0.70 (0.36-1.37) | 0.29 |
|  |  |  |  |
| **DCI definition** |  |  |  |
| Same as or close to gold standard definition | 42 | Ref |  |
| Defined other than gold standard | 14 | 1.11 (0.86-1.43) | 0.41 |
| **DCI**  **(Adjusted analysis)** |  |  |  |
| **Age** | 9 | 1.01 (0.92-1.10) | 0.83 |
| **Women (%)** | 9 | 1.04 (1.00-1.09) | **0.03** |
| **Study design** |  |  |  |
| Prospective | 6 | Ref |  |
| Retrospective | 2 | 1.16 (0.65-2.10) | 0.54 |
| **DCI definition** |  |  |  |
| Same as or close to gold standard definition | 6 | Ref |  |
| Defined other than gold standard | 2 | 0.92 (0.48-1.75) | 0.77 |

Supplementary Table-12 Analysis of Heterogeneity for Hydrocephalus studies

|  | **No. of studies** | **OR (95% CI)** | **P-value** |
| --- | --- | --- | --- |
| **SDHC**  **(Unadjusted analysis)** |  |  |  |
| **Age** | 51 | 0.97 (0.94-1.01) | 0.13 |
| **Women (%a)** | 53 | 0.99 (0.97-1.01) | 0.81 |
| **Study design** |  |  |  |
| Prospective | 11 | Ref |  |
| Retrospective | 37 | 1.16 (0.85-1.59) | 0.32 |
| Trial | 4 | 2.01 (1.22-3.30) | **0.007** |
| **SDHC**  **(Adjusted analysis)** |  |  |  |
| **Age** | 5 | 1.06 (0.46-2.42) | 0.82 |
| **Women (%a)** | 5 | 1.00 (0.89-1.12) | 0.89 |
|  |  |  |  |
| **Acute Hydrocephalus (Unadjusted analysis)** |  |  |  |
| **Age** | 6 | 1.04 (0.92-1.17) | 0.36 |
| **Women (%age)** | 9 | 0.99 (0.97-1.02) | 0.77 |

B


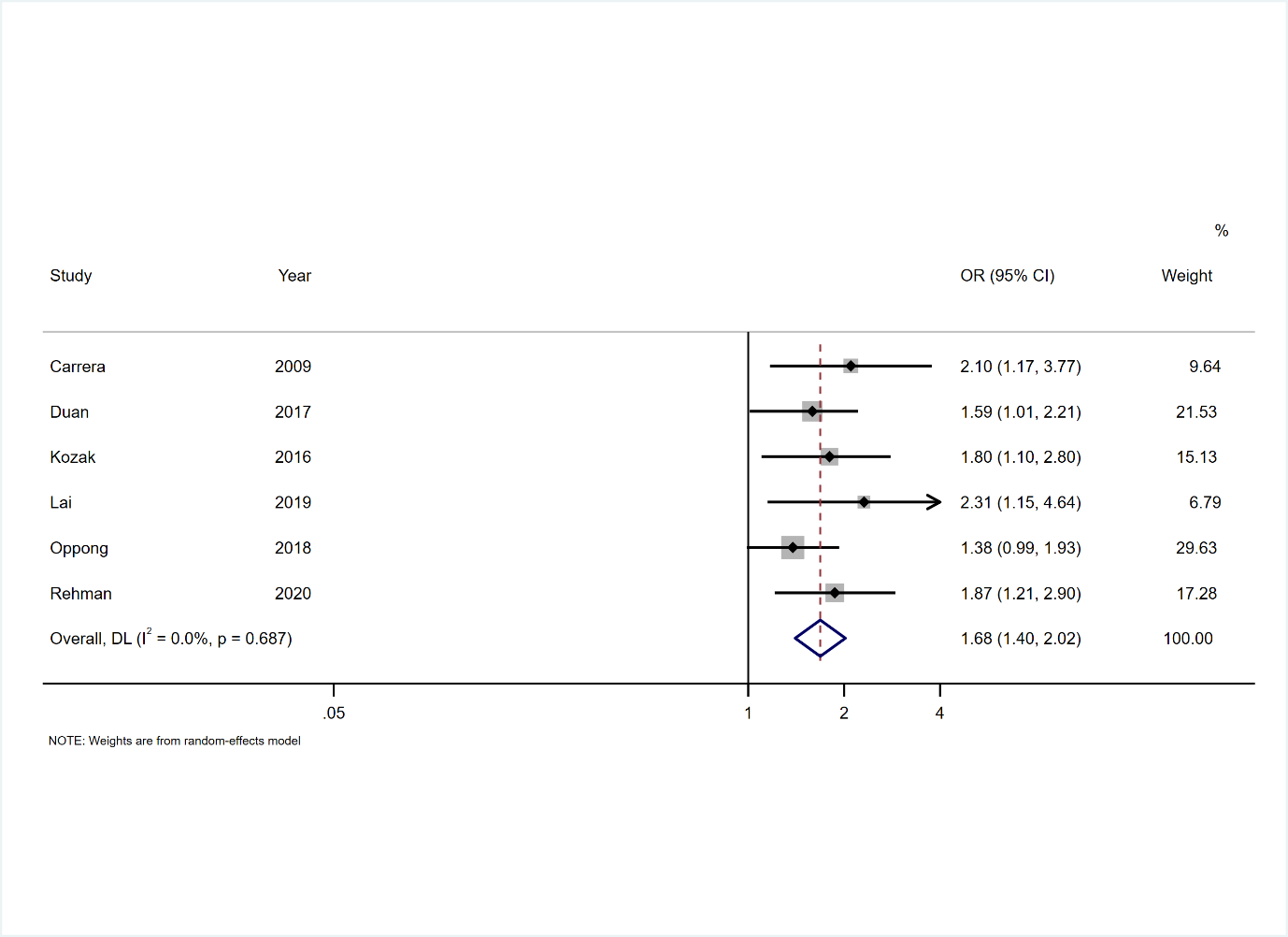

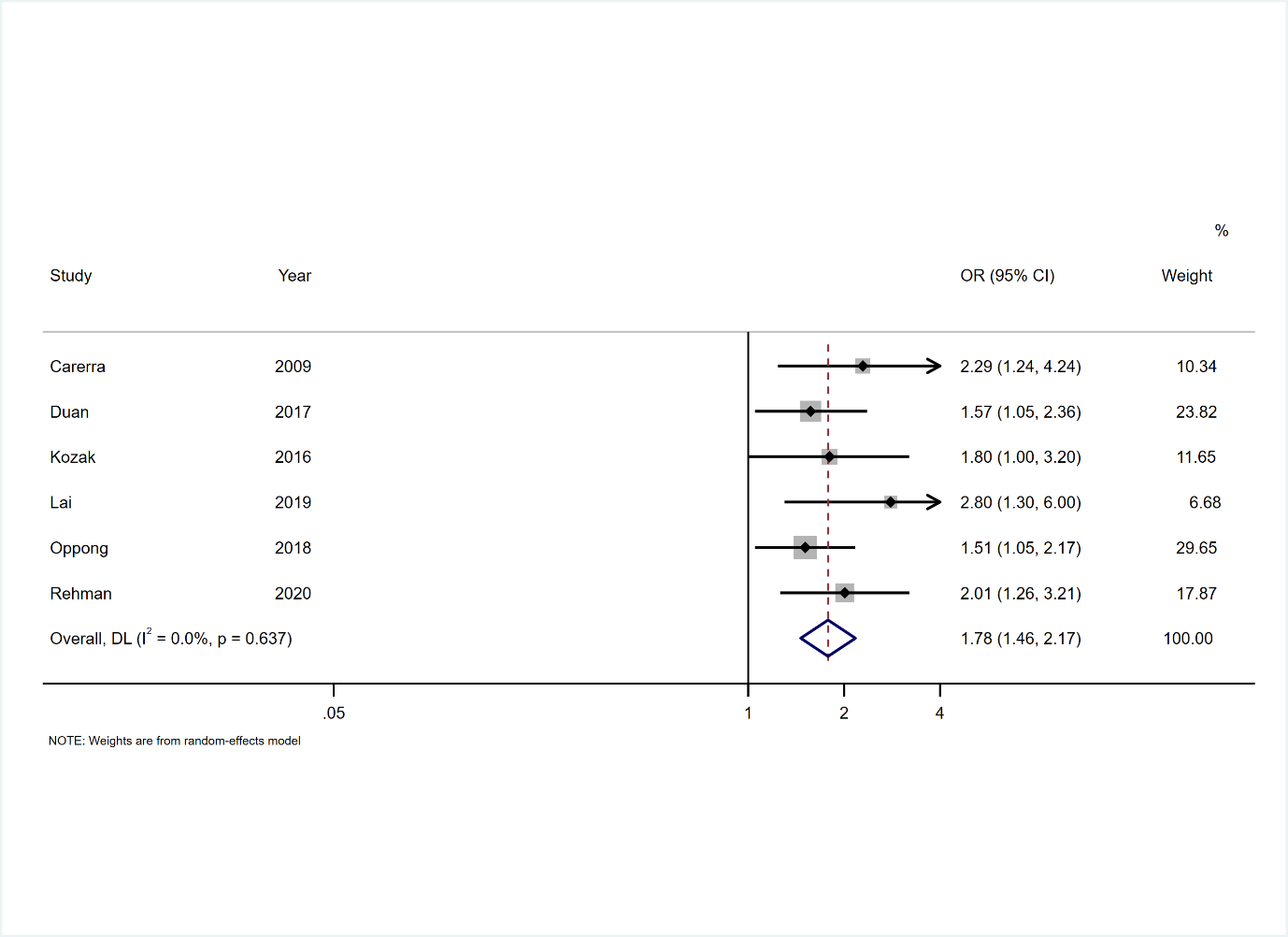


A

Supplementary figure-1 A comparison of pooled crude and adjusted estimates from similar studies (n=8) for DCI (A) pooled crude estimates (B) pooled adjusted estimates

**References:**

1. Aboul-Ela HM, El-Din AMS, Zaater AA, Shehab M, El Shahawy OA (2018) Predictors of shunt-dependent hydrocephalus following aneurysmal subarachnoid hemorrhage: a pilot study in a single Egyptian institute. The Egyptian journal of neurology, psychiatry and neurosurgery 54:1-6

2. Abulhasan YB, Jimenez JO, Teitelbaum J, Simoneau G, Angle MR (2020) Milrinone for refractory cerebral vasospasm with delayed cerebral ischemia. Journal of Neurosurgery 1:1-12

3. Ateia AM, Elbassiouny A, El-Nabi SH, Fahmy NA, Ibrahim MH, El-Garawani I, Geba KM, Khalaf M (2020) Predictive value of haptoglobin genotype as a risk of cerebral vasospasm after aneurysmal subarachnoid hemorrhage. Clinical Neurology and Neurosurgery 199:106296

4. Badjatia N, Seres D, Carpenter A, Schmidt JM, Lee K, Mayer SA, Claassen J, Connolly ES, Elkind MS (2012) Free fatty acids and delayed cerebral ischemia after subarachnoid hemorrhage. Stroke 43:691-696

5. Bae I-S, Yi H-J, Choi K-S, Chun H-J (2014) Comparison of incidence and risk factors for shunt-dependent hydrocephalus in aneurysmal subarachnoid hemorrhage patients. Journal of cerebrovascular and endovascular neurosurgery 16:78-84

6. Bakker AM, Dorhout Mees SM, Algra A, Rinkel GJ (2007) Extent of acute hydrocephalus after aneurysmal subarachnoid hemorrhage as a risk factor for delayed cerebral infarction. Stroke 38:2496-2499

7. Bjerkne Wenneberg S, Löwhagen Hendén PM, Oras J, Naredi S, Block L, Ljungqvist J, Odenstedt Hergès H (2020) Heart rate variability monitoring for the detection of delayed cerebral ischemia after aneurysmal subarachnoid hemorrhage. Acta Anaesthesiologica Scandinavica 64:945-952

8. Brandner S, Xu Y, Schmidt C, Emtmann I, Buchfelder M, Kleindienst A (2012) Shunt-dependent hydrocephalus following subarachnoid hemorrhage correlates with increased S100B levels in cerebrospinal fluid and serum. In: Intracranial Pressure and Brain Monitoring XIV. Springer, pp 217-220

9. Carrera E, Schmidt JM, Oddo M, Fernandez L, Claassen J, Seder D, Lee K, Badjatia N, Connolly Jr ES, Mayer SA (2009) Transcranial Doppler for predicting delayed cerebral ischemia after subarachnoid hemorrhage. Neurosurgery 65:316-324

10. Chan M, Alaraj A, Calderon M, Herrera SR, Gao W, Ruland S, Roitberg BZ (2009) Prediction of ventriculoperitoneal shunt dependency in patients with aneurysmal subarachnoid hemorrhage. Journal of neurosurgery 110:44-49

11. Chang JJ, Triano M, Corbin MJ, Desale S, Liu A-H, Felbaum DR, Mai JC, Armonda RA, Aulisi EF (2020) Transcranial Doppler velocity and associations with delayed cerebral ischemia in aneurysmal subarachnoid Hemorrhage. Journal of the neurological sciences 415:116934

12. Chang SI, Tsai MD, Yen DH-T, Hsieh C-T (2018) The clinical predictors of shunt-dependent hydrocephalus following aneurysmal subarachnoid hemorrhage. Turk Neurosurg 28:36-42

13. Charpentier C, Audibert G, Guillemin F, Civit T, Ducrocq X, Bracard S, Hepner H, Picard L, Laxenaire MC (1999) Multivariate analysis of predictors of cerebral vasospasm occurrence after aneurysmal subarachnoid hemorrhage. Stroke 30:1402-1408

14. Chen L, Pandey S, Shen R, Xu Y, Zhang Q (2021) Increased systemic immune-inflammation index is associated with delayed cerebral ischemia in aneurysmal subarachnoid hemorrhage patients. Frontiers in Neurology:1750

15. Crobeddu E, Mittal MK, Dupont S, Wijdicks EF, Lanzino G, Rabinstein AA (2012) Predicting the lack of development of delayed cerebral ischemia after aneurysmal subarachnoid hemorrhage. Stroke 43:697-701

16. Croci DM, Dalolio M, Aghlmandi S, Taub E, Rychen J, Chiappini A, Zumofen D, Guzman R, Mariani L, Roethlisberger M (2021) Early permanent cerebrospinal fluid diversion in aneurysmal subarachnoid hemorrhage: does a lower rate of nosocomial meningitis outweigh the risk of delayed cerebral vasospasm related morbidity? Neurological Research 43:40-53

17. Cuoco JA, Guilliams EL, Klein BJ, Benko MJ, Darden JA, Olasunkanmi AL, Witcher MR, Rogers CM, Marvin EA, Patel BM (2021) Neutrophil count on admission predicts acute symptomatic hydrocephalus after aneurysmal subarachnoid hemorrhage. World Neurosurgery 156:e338-e344

18. Czorlich P, Ricklefs F, Reitz M, Vettorazzi E, Abboud T, Regelsberger J, Westphal M, Schmidt NO (2015) Impact of intraventricular hemorrhage measured by Graeb and LeRoux score on case fatality risk and chronic hydrocephalus in aneurysmal subarachnoid hemorrhage. Acta neurochirurgica 157:409-415

19. Darkwah Oppong M, Iannaccone A, Gembruch O, Pierscianek D, Chihi M, Dammann P, Köninger A, Müller O, Forsting M, Sure U (2018) Vasospasm-related complications after subarachnoid hemorrhage: the role of patients’ age and sex. Acta neurochirurgica 160:1393-1400

20. De Marchis GM, Schaad C, Fung C, Beck J, Gralla J, Takala J, Jakob SM (2017) Gender-related differences in aneurysmal subarachnoid hemorrhage: a hospital based study. Clinical neurology and neurosurgery 157:82-87

21. de Oliveira JG, Beck J, Setzer M, Gerlach R, Vatter H, Seifert V, Raabe A (2007) Risk of shunt-dependent hydrocephalus after occlusion of ruptured intracranial aneurysms by surgical clipping or endovascular coiling: a single-institution series and meta-analysis. Neurosurgery 61:924-934

22. Dehdashti AR, Rilliet B, Rufenacht DA, de Tribolet N (2004) Shunt-dependent hydrocephalus after rupture of intracranial aneurysms: a prospective study of the influence of treatment modality. Journal of neurosurgery 101:402-407

23. Diesing D, Wolf S, Sommerfeld J, Sarrafzadeh A, Vajkoczy P, Dengler NF (2017) A novel score to predict shunt dependency after aneurysmal subarachnoid hemorrhage. Journal of neurosurgery 128:1273-1279

24. Ding C-Y, Cai H-P, Ge H-L, Yu L-H, Lin Y-X, Kang D-Z (2020) Is admission lipoprotein-associated phospholipase A2 a novel predictor of vasospasm and outcome in patients with aneurysmal subarachnoid hemorrhage? Neurosurgery 86:122-131

25. Dorai Z, Hynan LS, Kopitnik TA, Samson D (2003) Factors related to hydrocephalus after aneurysmal subarachnoid hemorrhage. Neurosurgery 52:763-771

26. Duan W, Pan Y, Wang C, Wang Y, Zhao X, Wang Y, Liu L, Investigators C (2018) Risk factors and clinical impact of delayed cerebral ischemia after aneurysmal subarachnoid hemorrhage: analysis from the China National Stroke Registry. Neuroepidemiology 50:128-136

27. Erixon HO, Sorteberg A, Sorteberg W, Eide PK (2014) Predictors of shunt dependency after aneurysmal subarachnoid hemorrhage: results of a single-center clinical trial. Acta neurochirurgica 156:2059-2069

28. Fan BB, Sun XC, Huang ZJ, Yang XM, Guo ZD, He ZH (2021) Hypoperfusion assessed by pressure reactivity index is associated with delayed cerebral ischemia after subarachnoid hemorrhage: an observational study. Chinese Neurosurgical Journal 7:1-9

29. Fang YJ, Mei SH, Lu JN, Chen YK, Chai ZH, Dong X, Araujo C, Reis C, Zhang JM, Chen S (2019) New risk score of the early period after spontaneous subarachnoid hemorrhage: For the prediction of delayed cerebral ischemia. CNS neuroscience & therapeutics 25:1173-1181

30. Fischer C, Goldberg J, Vulcu S, Wagner F, Schöni D, Söll N, Hänggi M, Schefold J, Fung C, Beck J (2019) Nimodipine-induced blood pressure changes can predict delayed cerebral ischemia. Frontiers in neurology 10:1161

31. Fukuda S, Koga Y, Fujita M, Suehiro E, Kaneda K, Oda Y, Ishihara H, Suzuki M, Tsuruta R (2019) Hyperoxemia during the hyperacute phase of aneurysmal subarachnoid hemorrhage is associated with delayed cerebral ischemia and poor outcome: a retrospective observational study. Journal of Neurosurgery 134:25-32

32. García-Armengol R, Puyalto P, Misis M, Julian JF, Rodríguez-Hernández A, Perez-Balaguero AC, Menendez B, Brugada F, Muñoz-Narbona L, Dominguez C (2021) Cerebrospinal fluid output as a risk factor of chronic hydrocephalus after aneurysmal subarachnoid hemorrhage. World neurosurgery 154:e572-e579

33. Graff-Radford NR, Torner J, Adams HP, Kassell NF (1989) Factors associated with hydrocephalus after subarachnoid hemorrhage: a report of the Cooperative Aneurysm Study. Archives of neurology 46:744-752

34. Guan J, Karsy M, Brock A, Couldwell WT (2016) The Utility of Ankle-Brachial Index as a Predictor of Delayed Cerebral Ischemia in Aneurysmal Subarachnoid Hemorrhage. World neurosurgery 89:139-146

35. Hamdan A, Barnes J, Mitchell P (2014) Subarachnoid hemorrhage and the female sex: analysis of risk factors, aneurysm characteristics, and outcomes. Journal of neurosurgery 121:1367-1373

36. Hao X, Wei D (2019) The risk factors of shunt-dependent hydrocephalus after subarachnoid space hemorrhage of intracranial aneurysms. Medicine 98

37. Heit JJ, Wintermark M, Martin BW, Zhu G, Marks MP, Zaharchuk G, Dodd RL, Do HM, Steinberg GK, Lansberg MG (2018) Reduced intravoxel incoherent motion microvascular perfusion predicts delayed cerebral ischemia and vasospasm after aneurysm rupture. Stroke 49:741-745

38. Hirashima Y, Hamada H, Hayashi N, Kuwayama N, Origasa H, Endo S (2003) Independent predictors of late hydrocephalus in patients with aneurysmal subarachnoid hemorrhage–analysis by multivariate logistic regression model. Cerebrovascular Diseases 16:205-210

39. Hirashima Y, Kurimoto M, Hori E, Origasa H, Endo S (2005) Lower incidence of symptomatic vasospasm after subarachnoid hemorrhage owing to ruptured vertebrobasilar aneurysms. Neurosurgery 57:1110-1116

40. Hop J, Rinkel G, Algra A, Van Gijn J (1999) Initial loss of consciousness and risk of delayed cerebral ischemia after aneurysmal subarachnoid hemorrhage. Stroke 30:2268-2271

41. Hu P, Yang X, Li Y, Deng G, Xu Y, Ye L, Qi Y, Zong Z, Chen Q (2022) Predictive effects of admission white blood cell counts and hounsfield unit values on delayed cerebral ischemia after aneurysmal subarachnoid hemorrhage. Clinical Neurology and Neurosurgery 212:107087

42. Jabbarli R, Pierscianek D, Rölz R, Oppong MD, Kaier K, Shah M, Taschner C, Mönninghoff C, Urbach H, Beck J (2019) Endovascular treatment of cerebral vasospasm after subarachnoid hemorrhage: more is more. Neurology 93:e458-e466

43. Jartti P, Karttunen A, Jartti A, Ukkola V, Sajanti J, Pyhtinen J (2004) Factors related to acute hydrocephalus after subarachnoid hemorrhage. Acta radiologica 45:333-339

44. Jeong TS, Yoo CJ, Kim WK, Yee GT, Kim EY, Kim MJ (2017) Factors related to the development of shunt-dependent hydrocephalus following subarachnoid hemorrhage in the elderly. Turk Neurosurg:19752-19716.19751

45. Jovanović I, Nemir J, Gardijan D, Milošević M, Poljaković Z, Klarica M, Ozretić D, Radoš M (2021) Transient acute hydrocephalus after aneurysmal subarachnoid hemorrhage and aneurysm embolization: a single-center experience. Neuroradiology 63:2111-2119

46. Kale SP, Edgell RC, Alshekhlee A, Haghighi AB, Sweeny J, Felton J, Kitchener J, Vora N, Bieneman BK, Cruz-Flores S (2013) Age-associated vasospasm in aneurysmal subarachnoid hemorrhage. Journal of Stroke and Cerebrovascular Diseases 22:22-27

47. Kasius K, Frijns C, Algra A, Rinkel G (2010) Association of platelet and leukocyte counts with delayed cerebral ischemia in aneurysmal subarachnoid hemorrhage. Cerebrovascular Diseases 29:576-583

48. Kaur G, Damodara N, Feldstein E, Dominguez J, Huang KT, Ogulnick JV, Nuoman R, Khandelwal P, El-Ghanem M, Gupta G (2021) Relation between brain natriuretic peptide and delayed cerebral ischemia in patients with aneurysmalsubarachnoid hemorrhage. Clinical Neurology and Neurosurgery 211:107031

49. Kawabata Y, Horikawa F, Ueno Y, Sawada M, Isaka F, Miyake H (2011) Clinical predictors of delayed cerebral ischemia after subarachnoid hemorrhage: first experience with coil embolization in the management of ruptured cerebral aneurysms. Journal of neurointerventional surgery 3:344-347

50. Kawano A, Sugimoto K, Nomura S, Inoue T, Kawano R, Oka F, Sadahiro H, Ishihara H, Suzuki M (2021) Association between spreading depolarization and delayed cerebral ischemia after subarachnoid hemorrhage: post hoc analysis of a randomized trial of the effect of cilostazol on delayed cerebral ischemia. Neurocritical Care 35:91-99

51. Kim JH, Kim JH, Kang HI, Kim DR, Moon BG, Kim JS (2019) Risk factors and preoperative risk scoring system for shunt-dependent hydrocephalus following aneurysmal subarachnoid hemorrhage. Journal of Korean Neurosurgical Society 62:643

52. Kim SH, Chung P-W, Won YS, Kwon YJ, Shin HC, Choi CS (2012) Effect of cisternal drainage on the shunt dependency following aneurysmal subarachnoid hemorrhage. Journal of Korean Neurosurgical Society 52:441

53. Ko S-B, Choi HA, Carpenter AM, Helbok R, Schmidt JM, Badjatia N, Claassen J, Connolly ES, Mayer SA, Lee K (2011) Quantitative analysis of hemorrhage volume for predicting delayed cerebral ischemia after subarachnoid hemorrhage. Stroke 42:669-674

54. Koyanagi M, Fukuda H, Saiki M, Tsuji Y, Lo B, Kawasaki T, Ioroi Y, Fukumitsu R, Ishibashi R, Oda M (2018) Effect of choice of treatment modality on the incidence of shunt-dependent hydrocephalus after aneurysmal subarachnoid hemorrhage. Journal of neurosurgery 130:949-955

55. Kozak N, Bereczki D, Szabo S (2016) Predictors of symptomatic vasospasm after subarachnoid hemorrhage: a single center study of 457 consecutive cases. Turk Neurosurg 26:545-549

56. Kramer AH, Mikolaenko I, Deis N, Dumont AS, Kassell NF, Bleck TP, Nathan BA (2010) Intraventricular hemorrhage volume predicts poor outcomes but not delayed ischemic neurological deficits among patients with ruptured cerebral aneurysms. Neurosurgery 67:1044-1053

57. Kwon J-H, Sung S-K, Song Y-J, Choi H-J, Huh J-T, Kim H-D (2008) Predisposing factors related to shunt-dependent chronic hydrocephalus after aneurysmal subarachnoid hemorrhage. Journal of Korean Neurosurgical Society 43:177

58. Lai PMR, Gormley WB, Patel N, Frerichs KU, Aziz-Sultan MA, Du R (2019) Age-dependent radiographic vasospasm and delayed cerebral ischemia in women after aneurysmal subarachnoid hemorrhage. World neurosurgery 130:e230-e235

59. Lasner TM, Weil RJ, Riina HA, King JT, Zager EL, Raps EC, Flamm ES (1997) Cigarette smoking—induced increase in the risk of symptomatic vasospasm after aneurysmal subarachnoid hemorrhage. Journal of neurosurgery 87:381-384

60. Lee H, Perry JJ, English SW, Alkherayf F, Joseph J, Nobile S, Zhou LL, Lesiuk H, Moulton R, Agbi C (2018) Clinical prediction of delayed cerebral ischemia in aneurysmal subarachnoid hemorrhage. Journal of neurosurgery 130:1914-1921

61. Lenski M, Biczok A, Huge V, Forbrig R, Briegel J, Tonn J-C, Thon N (2019) Role of cerebrospinal fluid markers for predicting shunt-dependent hydrocephalus in patients with subarachnoid hemorrhage and external ventricular drain placement. World neurosurgery 121:e535-e542

62. Lewis A, Irvine H, Ogilvy C, Kimberly WT (2015) Predictors for delayed ventriculoperitoneal shunt placement after external ventricular drain removal in patients with subarachnoid hemorrhage. British journal of neurosurgery 29:219-224

63. Liang H, Gui B, Gao A, Meng X, Li C, Ma L, Rong Y, Zhang H, Zhang J, Luo X (2022) The MAI Score: A Novel Score to Early Predict Shunt-dependent Hydrocephalus in Patients With Aneurysmal Subarachnoid Hemorrhage After Surgery. Clinical Neurology and Neurosurgery:107317

64. Lin C-L, Kwan A-L, Howng S-L (1999) Acute hydrocephalus and chronic hydrocephalus with the need of postoperative shunting after aneurysmal subarachnoid hemorrhage. The Kaohsiung journal of medical sciences 15:137-145

65. Megjhani M, Terilli K, Weiss M, Savarraj J, Chen LH, Alkhachroum A, Roh DJ, Agarwal S, Connolly Jr ES, Velazquez A (2021) Dynamic detection of delayed cerebral ischemia: a study in 3 centers. Stroke 52:1370-1379

66. Mehta V, Holness R, Connolly K, Walling S, Hall R (1996) Acute hydrocephalus following aneurysmal subarachnoid hemorrhage. Canadian journal of neurological sciences 23:40-45

67. Mijderwijk H-J, Fischer I, Zhivotovskaya A, Bostelmann R, Steiger H-J, Cornelius JF, Petridis AK (2019) Prognostic model for chronic shunt-dependent hydrocephalus after aneurysmal subarachnoid hemorrhage. World neurosurgery 124:e572-e579

68. Mijiti M, Mijiti P, Axier A, Amuti M, Guohua Z, Xiaojiang C, Kadeer K, Xixian W, Geng D, Maimaitili A (2016) Incidence and predictors of angiographic vasospasm, symptomatic vasospasm and cerebral infarction in Chinese patients with aneurysmal subarachnoid hemorrhage. PLoS One 11:e0168657

69. Moskowitz SI, Ahrens C, Provencio JJ, Chow M, Rasmussen PA (2009) Prehemorrhage statin use and the risk of vasospasm after aneurysmal subarachnoid hemorrhage. Surg Neurol 71:311-318. doi:10.1016/j.surneu.2007.12.027

70. Motiei-Langroudi R, Adeeb N, Foreman PM, Harrigan MR, Fisher 3rd WS, Vyas NA, Lipsky RH, Walters BC, Tubbs SR, Shoja MM (2017) Predictors of shunt insertion in aneurysmal subarachnoid hemorrhage. World neurosurgery 98:421-426

71. Nakatsuka Y, Kawakita F, Yasuda R, Umeda Y, Toma N, Sakaida H, Suzuki H (2017) Preventive effects of cilostazol against the development of shunt-dependent hydrocephalus after subarachnoid hemorrhage. Journal of neurosurgery 127:319-326

72. Nam K-H, Hamm I-S, Kang D-H, Park J, Kim Y-S (2010) Risk of shunt dependent hydrocephalus after treatment of ruptured intracranial aneurysms: surgical clipping versus endovascular coiling according to fisher grading system. Journal of Korean Neurosurgical Society 48:313

73. Naraoka M, Matsuda N, Shimamura N, Ohkuma H (2022) Role of microcirculatory impairment in delayed cerebral ischemia and outcome after aneurysmal subarachnoid hemorrhage. Journal of Cerebral Blood Flow & Metabolism 42:186-196

74. Nassar HGE, Ghali AA, Bahnasy WS, Elawady MM (2019) Vasospasm following aneurysmal subarachnoid hemorrhage: prediction, detection, and intervention. The Egyptian journal of neurology, psychiatry and neurosurgery 55:1-6

75. Neidert MC, Maldaner N, Stienen MN, Roethlisberger M, Zumofen DW, D’Alonzo D, Marbacher S, Maduri R, Hostettler IC, Schatlo B (2018) The barrow neurological institute grading scale as a predictor for delayed cerebral ischemia and outcome after aneurysmal subarachnoid hemorrhage: data from a nationwide patient registry (Swiss SOS). Neurosurgery 83:1286-1293

76. Nguyen AM, Williamson CA, Pandey AS, Sheehan KM, Rajajee V (2021) Screening Computed Tomography Angiography to Identify Patients at Low Risk for Delayed Cerebral Ischemia Following Aneurysmal Subarachnoid Hemorrhage. Frontiers in Neurology 12

77. O'Kelly CJ, Kulkarni AV, Austin PC, Urbach D, Wallace MC (2009) Shunt-dependent hydrocephalus after aneurysmal subarachnoid hemorrhage: incidence, predictors, and revision rates. Journal of neurosurgery 111:1029-1035

78. Paisan GM, Ding D, Starke RM, Crowley RW, Liu KC (2018) Shunt-dependent hydrocephalus after aneurysmal subarachnoid hemorrhage: predictors and long-term functional outcomes. Neurosurgery 83:393-402

79. Park S, Megjhani M, Frey H-P, Grave E, Wiggins C, Terilli KL, Roh DJ, Velazquez A, Agarwal S, Connolly Jr ES (2019) Predicting delayed cerebral ischemia after subarachnoid hemorrhage using physiological time series data. Journal of clinical monitoring and computing 33:95

80. Park YK, Yi H-J, Choi K-S, Lee Y-J, Chun H-J, Kwon SM, Kim D-W (2018) Predicting factors for shunt-dependent hydrocephalus in patients with aneurysmal subarachnoid hemorrhage. Acta neurochirurgica 160:1407-1413

81. Perry A, Graffeo C, Kleinstern G, Carlstrom L, Link M, Rabinstein A (2020) Quantitative modeling of external ventricular drain output to predict shunt dependency in aneurysmal subarachnoid hemorrhage: cohort study. Neurocritical care 33:218-229

82. Pinggera D, Kerschbaumer J, Petr O, Ortler M, Thomé C, Freyschlag CF (2017) The volume of the third ventricle as a prognostic marker for shunt dependency after aneurysmal subarachnoid hemorrhage. World neurosurgery 108:107-111

83. Platz J, Güresir E, Wagner M, Seifert V, Konczalla J (2017) Increased risk of delayed cerebral ischemia in subarachnoid hemorrhage patients with additional intracerebral hematoma. Journal of neurosurgery 126:504-510

84. Qureshi AI, Sung GY, Razumovsky AY, Lane K, Straw RN, Ulatowski JA (2000) Early identification of patients at risk for symptomatic vasospasm after aneurysmal subarachnoid hemorrhage. Critical care medicine 28:984-990

85. Raatikainen E, Vahtera A, Kuitunen A, Junttila E, Huhtala H, Ronkainen A, Pyysalo L, Kiiski H (2021) Prognostic value of the 2010 consensus definition of delayed cerebral ischemia after aneurysmal subarachnoid hemorrhage. Journal of the Neurological Sciences 420:117261

86. Rehman S, Chandra RV, Zhou K, Tan D, Lai L, Asadi H, Froelich J, Thani N, Nichols L, Blizzard L (2020) Sex differences in aneurysmal subarachnoid haemorrhage (aSAH): aneurysm characteristics, neurological complications, and outcome. Acta Neurochirurgica 162:2271-2282

87. Rinaldo L, Rabinstein AA, Lanzino G (2019) Increased body mass index associated with reduced risk of delayed cerebral ischemia and subsequent infarction after aneurysmal subarachnoid hemorrhage. Neurosurgery 84:1035-1042

88. Rincon F, Gordon E, Starke RM, Buitrago MM, Fernandez A, Schmidt JM, Claassen J, Wartenberg KE, Frontera J, Seder DB (2010) Predictors of long-term shunt-dependent hydrocephalus after aneurysmal subarachnoid hemorrhage. Journal of neurosurgery 113:774-780

89. Rios JMV, Sanchez-Aguilar M, Kretschmer T, Heinen C, Govea FAM, Juan S-RJ, Schmidt T (2018) Predictors of hydrocephalus as a complication of non-traumatic subarachnoid hemorrhage: a retrospective observational cohort study in 107 patients. Patient safety in surgery 12:1-8

90. Ritzenthaler T, Gobert F, Bouchier B, Dailler F (2021) Amount of blood during the subacute phase and clot clearance rate as prognostic factors for delayed cerebral ischemia after aneurysmal subarachnoid hemorrhage. Journal of Clinical Neuroscience 87:74-79

91. Sanelli P, Anumula N, Johnson C, Comunale J, Tsiouris A, Riina H, Segal A, Stieg P, Zimmerman R, Mushlin A (2013) Evaluating CT perfusion using outcome measures of delayed cerebral ischemia in aneurysmal subarachnoid hemorrhage. American journal of neuroradiology 34:292-298

92. Saripalli M, Tan D, Chandra RV, Lai LT (2021) Predictive relevance of early temperature elevation on the risk of delayed cerebral ischemia development following aneurysmal subarachnoid hemorrhage. World Neurosurgery 150:e474-e481

93. Schembri M, Verbaan D, Emmer BJ, Coert BA, Majoie CB, Vandertop WP, van den Berg R (2021) Cerebral circulation time on DSA during endovascular treatment in WFNS grade I aneurysmal SAH patients—a predictor of DCI? Neuroradiology 63:2131-2138

94. Sheehan JP, Polin RS, Sheehan JM, Baskaya MK, Kassell NF (1999) Factors associated with hydrocephalus after aneurysmal subarachnoid hemorrhage. Neurosurgery 45:1120-1128

95. Sugawara T, Maehara T, Nariai T, Aoyagi M, Ohno K (2014) Independent predictors of shunt-dependent normal pressure hydrocephalus after aneurysmal subarachnoid hemorrhage. Journal of neurosurgical sciences 60:154-158

96. Talbot-Stetsko HK, Raue KD, Aaron BL, Adapa AR, Altshuler DB, Srinivasan S, Pandey AS, Maher CO, Hollon TC, Khalsa SSS (2022) Ventricular Volume Change as a Predictor of Shunt-Dependent Hydrocephalus in Aneurysmal Subarachnoid Hemorrhage. World Neurosurgery 157:e57-e65

97. Tapaninaho A, Hernesniemi J, Vapalahti M, Niskanen M, Kari A, Luukkonen M, Puranen M (1993) Shunt-dependent hydrocephalus after subarachnoid haemorrhage and aneurysm surgery: timing of surgery is not a risk factor. Acta neurochirurgica 123:118-124

98. Tso MK, Ibrahim GM, Macdonald RL (2016) Predictors of shunt-dependent hydrocephalus following aneurysmal subarachnoid hemorrhage. World neurosurgery 86:226-232

99. van der Steen WE, Marquering HA, Boers AM, Ramos LA, van den Berg R, Vergouwen MD, Majoie CB, Coert BA, Vandertop WP, Verbaan D (2019) Predicting delayed cerebral ischemia with quantified aneurysmal subarachnoid blood volume. World neurosurgery 130:e613-e619

100. Van Donkelaar CE, Dijkland SA, Van Den Bergh WM, Bakker J, Dippel DW, Nijsten MW, van der Jagt M (2016) Early circulating lactate and glucose levels after aneurysmal subarachnoid hemorrhage correlate with poor outcome and delayed cerebral ischemia: a two-center cohort study. Critical care medicine 44:966-972

101. Varelas P, Helms A, Sinson G, Spanaki M, Hacein-Bey L (2006) Clipping or coiling of ruptured cerebral aneurysms and shunt-dependent hydrocephalus. Neurocritical care 4:223-228

102. Vermeij F, Hasan D, Vermeulen M, Tanghe H, Van Gijn J (1994) Predictive factors for deterioration from hydrocephalus after subarachnoid hemorrhage. Neurology 44:1851-1851

103. Walcott BP, Iorgulescu JB, Stapleton CJ, Kamel H (2015) Incidence, timing, and predictors of delayed shunting for hydrocephalus after aneurysmal subarachnoid hemorrhage. Neurocritical care 23:54-58

104. Wang K-C, Tang S-C, Lee J-E, Jeng J-S, Lai D-M, Huang S-J, Hsieh S-T, Tu Y-K (2015) Intrathecal lactate predicting hydrocephalus after aneurysmal subarachnoid hemorrhage. journal of surgical research 199:523-528

105. Wang Y-M, Lin Y-J, Chuang M-J, Lee T-H, Tsai N-W, Cheng B-C, Lin W-C, Su BY-J, Yang T-M, Chang W-N (2012) Predictors and outcomes of shunt-dependent hydrocephalus in patients with aneurysmal sub-arachnoid hemorrhage. BMC surgery 12:1-8

106. Wessell AP, Kole MJ, Cannarsa G, Oliver J, Jindal G, Miller T, Gandhi D, Parikh G, Badjatia N, Aldrich EF (2018) A sustained systemic inflammatory response syndrome is associated with shunt-dependent hydrocephalus after aneurysmal subarachnoid hemorrhage. Journal of neurosurgery 130:1984-1991

107. Woernle CM, Winkler KM, Burkhardt J-K, Haile SR, Bellut D, Neidert MC, Bozinov O, Krayenbühl N, Bernays R-L (2013) Hydrocephalus in 389 patients with aneurysm-associated subarachnoid hemorrhage. Journal of clinical neuroscience 20:824-826

108. Won YD, Kim JM, Cheong JH, Ryu JI, Yi H-J, Han M-H (2021) Effect of osteoporotic condition on ventriculomegaly and shunt-dependent hydrocephalus after subarachnoid hemorrhage. Stroke 52:994-1003

109. Wostrack M, Reeb T, Martin J, Kehl V, Shiban E, Preuss A, Ringel F, Meyer B, Ryang Y-M (2014) Shunt-dependent hydrocephalus after aneurysmal subarachnoid hemorrhage: the role of intrathecal interleukin-6. Neurocritical care 21:78-84

110. Wu Y, He Q, Wei Y, Zhu J, He Z, Zhang X, Guo Z, Xu R, Cheng C, Huang Z (2019) The association of neutrophil-to-lymphocyte ratio and delayed cerebral ischemia in patients with aneurysmal subarachnoid hemorrhage: possible involvement of cerebral blood perfusion. Neuropsychiatric disease and treatment 15:1001

111. Yang T-C, Chang CH, Liu Y-T, Chen Y-L, Tu P-H, Chen H-C (2013) Predictors of shunt-dependent chronic hydrocephalus after aneurysmal subarachnoid haemorrhage. European neurology 69:296-303

112. Yang X, Peng J, Pang J, Wan W, Zhong C, Peng T, Bao K, Jiang Y (2020) The association between serum macrophage migration inhibitory factor and delayed cerebral ischemia after aneurysmal subarachnoid hemorrhage. Neurotoxicity research 37:397-405

113. Yang Y-C, Yin C-H, Chen K-T, Lin P-C, Lee C-C, Liao W-C, Chen J-S (2021) Prognostic nomogram of predictors for shunt-dependent hydrocephalus in patients with aneurysmal subarachnoid hemorrhage receiving external ventricular drain insertion: a single-center experience and narrative review. World Neurosurgery 150:e12-e22

114. Yao P-S, Chen G-R, Zheng S-F, Kang D-Z (2017) Predictors of postoperative cerebral ischemia in patients with ruptured anterior communicating artery aneurysms. World neurosurgery 103:241-247

115. Yoneda H, Nakamura T, Shirao S, Tanaka N, Ishihara H, Suehiro E, Koizumi H, Isotani E, Suzuki M (2013) Multicenter prospective cohort study on volume management after subarachnoid hemorrhage: hemodynamic changes according to severity of subarachnoid hemorrhage and cerebral vasospasm. Stroke 44:2155-2161

116. Yu H, Zhan R, Wen L, Shen J, Fan Z (2014) The relationship between risk factors and prognostic factors in patients with shunt-dependent hydrocephalus after aneurysmal subarachnoid hemorrhage. Journal of Craniofacial Surgery 25:902-906

117. Zaidi HA, Montoure A, Elhadi A, Nakaji P, McDougall CG, Albuquerque FC, Spetzler RF, Zabramski JM (2015) Long-term functional outcomes and predictors of shunt-dependent hydrocephalus after treatment of ruptured intracranial aneurysms in the BRAT trial: revisiting the clip vs coil debate. Neurosurgery 76:608-615

118. Zhang X, Liu Y, Zhang S, Wang C, Zou C, Li A (2021) Neutrophil-to-albumin ratio as a biomarker of delayed cerebral ischemia after aneurysmal subarachnoid hemorrhage. World Neurosurgery 147:e453-e458

119. Zhang Y, Zheng S, Wang H, Chen G, Li C, Lin Y, Yao P, Kang D (2022) Admission Lower Serum Phosphate Ion Levels Predict Acute Hydrocephalus of Aneurysmal Subarachnoid Hemorrhage. Frontiers in Neurology 12:759963

120. Zhao L, Cheng C, Peng L, Zuo W, Xiong D, Zhang L, Mao Z, Wu X, Jiang X, Wang P (2022) Alcohol Abuse Associated With Increased Risk of Angiographic Vasospasm and Delayed Cerebral Ischemia in Patients With Aneurysmal Subarachnoid Hemorrhage Requiring Mechanical Ventilation. Frontiers in Cardiovascular Medicine 9
